# The Safety of Artemisinin Derivatives for the Treatment of Malaria in the 2nd or 3rd Trimester of Pregnancy: A Systematic Review and Meta-Analysis

**DOI:** 10.1371/journal.pone.0164963

**Published:** 2016-11-08

**Authors:** Stephanie D. Kovacs, Anna Maria van Eijk, Esperanca Sevene, Stephanie Dellicour, Noel S. Weiss, Scott Emerson, Richard Steketee, Feiko O. ter Kuile, Andy Stergachis

**Affiliations:** 1 Department of Epidemiology, University of Washington, Seattle, WA, United States of America; 2 Liverpool School of Tropical Medicine, Liverpool, United Kingdom; 3 Manhiça Health Research Centre, Faculty of Medicine, Eduardo Mondlane University, Maputo, Mozambique; 4 Department of Biostatistics, University of Washington, Seattle, WA, United States of America; 5 PATH, Seattle, WA, United States of America; 6 Kenya Medical Research Institute (KEMRI) Centre for Global Health, Kisumu, Kenya; 7 Department of Global Health, University of Washington, Seattle, WA, United States of America; 8 Department of Pharmacy, University of Washington, Seattle, WA, United States of America; Centers for Disease Control and Prevention, UNITED STATES

## Abstract

Given the high morbidity for mother and fetus associated with malaria in pregnancy, safe and efficacious drugs are needed for treatment. Artemisinin derivatives are the most effective antimalarials, but are associated with teratogenic and embryotoxic effects in animal models when used in early pregnancy. However, several organ systems are still under development later in pregnancy. We conducted a systematic review and meta-analysis of the occurrence of adverse pregnancy outcomes among women treated with artemisinins monotherapy or as artemisinin-based combination therapy during the 2nd or 3rd trimesters relative to pregnant women who received non-artemisinin antimalarials or none at all. Pooled odds ratio (POR) were calculated using Mantel-Haenszel fixed effects model with a 0.5 continuity correction for zero events. Eligible studies were identified through Medline, Embase, and the Malaria in Pregnancy Consortium Library. Twenty studies (11 cohort studies and 9 randomized controlled trials) contributed to the analysis, with 3,707 women receiving an artemisinin, 1,951 a non-artemisinin antimalarial, and 13,714 no antimalarial. The PORs (95% confidence interval (CI)) for stillbirth, fetal loss, and congenital anomalies when comparing artemisinin versus quinine were 0.49 (95% CI 0.24–0.97, I^2^ = 0%, 3 studies); 0.58 (95% CI 0.31–1.16, I^2^ = 0%, 6 studies); and 1.00 (95% CI 0.27–3.75, I^2^ = 0%, 3 studies), respectively. The PORs comparing artemisinin users to pregnant women who received no antimalarial were 1.13 (95% CI 0.77–1.66, I^2^ = 86.7%, 3 studies); 1.10 (95% CI 0.79–1.54, I^2^ = 0%, 4 studies); and 0.79 (95% CI 0.37–1.67, I^2^ = 0%, 3 studies) for miscarriage, stillbirth and congenital anomalies respectively. Treatment with artemisinin in 2^nd^ and 3^rd^ trimester was not associated with increased risks of congenital malformations or miscarriage and may be was associated with a reduced risk of stillbirths compared to quinine. This study updates the reviews conducted by the WHO in 2002 and 2006 and supports the current WHO malaria treatment guidelines malaria in pregnancy.

## Introduction

Given the high morbidity and mortality associated with malaria in pregnancy, safe and effective drugs are needed for treatment and prevention [1]. However, there is limited information on the safety profile of most antimalarials when used during pregnancy, in part because few clinical trials enroll pregnant women and there are few examples of systematic approaches to pregnancy pharmacovigilance [2,3].

The World Health Organization (WHO) currently recommends the use of artemisinin-based combination therapy (ACT) for the treatment of uncomplicated malaria in adults, children and in pregnant women in the 2nd or 3rd trimester [4]. Seven days of quinine with clindamycin is recommended for uncomplicated malaria in the first trimester of pregnancy [5]. The reports from relatively small observational studies and randomized controlled trials (RCTs) have not identified an increased risk of miscarriage or stillbirth after receipt of artemisinins during pregnancy, compared to women receiving non-artemisinin antimalarials or to pregnant women who did not receive any antimalarials [6–12].

The WHO recommends the use of ACTs in the first trimester if this is the only treatment immediately available, or if treatment with 7-day quinine plus clindamycin fails or if there is uncertainty about adherence to a 7-day treatment [13]. Studies of rats, rabbits, and primates have reported the artemisinin class of antimalarial drugs to be embryotoxic and teratogenic [2]. These animal studies suggest that the etiologically relevant time period for exposure in humans is within the first trimester. Several organs are still under development in the 2^nd^ trimester and therefore sensitive to teratogens, including the uterus (18 weeks), brain (until birth), eyes (24–36 weeks), and ears (18 weeks) [14], although animal data did not observe these types of anomalies after artemisinin exposure [15]. Nevertheless, animal data do not always mirror the effects observed in humans.

We conducted a systematic review and meta-analysis examining the risk of adverse pregnancy outcomes associated with 2nd or 3rd trimester use of an artemisinin, compared with the experience of pregnant women treated with other antimalarial therapies and pregnant women without malaria and therefore no exposure to an antimalarial.

## Methods

This analysis was completed in accordance with the PRISMA guidelines (S1 and S2 Figs) [16]. The safety outcomes assessed were miscarriage (<28 weeks), stillbirth (>/ = 28 weeks), fetal loss (composite of miscarriage or stillbirth), and congenital anomalies.

### Search Strategy

We conducted an electronic search of Medline, Embase, and the Malaria in Pregnancy Library as of January 12, 2015, and the Medline search was updated on June 15, 2015, using the Patient, Intervention, Comparator, Outcome, Timing and Setting (PICOTS) framework (S1 Fig) [17,18]. We also searched 'gray literature' databases, conference abstracts and manually reviewed reference lists of selected publications.

### Inclusion/exclusion criteria

Studies fulfilling the following criteria were eligible for inclusion: prospective cohort studies or RCTs enrolling women of child bearing age (WOCBA) or pregnant women of any gestational age who received an artemisinin in the 2^nd^ or 3^rd^ trimester either as a monotherapy or as an ACT for the treatment of *P*. *falciparum*, *P*. *vivax* or mixed infections, and with information available on fetal loss and/or congenital anomalies, and information on timing of exposure in pregnancy (e.g. 2nd or 3rd trimester). Study exclusion criteria were case-control studies and cross-sectional surveys, studies not involving artemisinins treatment, studies reporting only 1^st^ trimester artemisinin exposures, and studies that did not report pregnancy outcomes. In studies that reported exposures in both first trimester and 2^nd^ or 3^rd^ trimester, the pregnancies with first trimester exposures were excluded from the analysis.

### Data Extraction

Two reviewers (SK and AE) independently screened titles and abstracts of all citations to identify potentially eligible studies (first screen). The second screen consisted of full-text review of studies selected by either reviewer in the first screen with agreement required for final study eligibility and inclusion in the systematic review. Any disagreements on study inclusion were resolved by discussions between the reviewers. The two reviewers independently extracted data using a standardized form and resolved any discrepancies by consensus. The primary data abstracted included number, type, and timing of antimalarial drug exposures; patient characteristics including age, parity, parasitemia level, and HIV prevalence; and pregnancy outcomes including the number of miscarriages, stillbirths, and congenital anomalies reported by exposure. In addition we created a composite outcome of “any fetal loss” which combined any miscarriage or stillbirth for studies that did not clearly delineate the number of exposures in the 2^nd^ and 3^rd^ trimester.

### Bias assessment

To assess the possibility of bias we applied the Cochrane Collaboration’s tool to score RCT studies as having high, low, or unclear risk of bias [19]. The Newcastle Ottawa scale was used to evaluate cohort studies for selection bias, comparability, and assessment of the outcome [20].

### Exposure groups

The main exposure group of interest consisted of pregnant women who received artemisinin treatment in the 2^nd^ or 3^rd^ trimester. The comparison groups consisted of the following other exposure groups 1) treatment with oral quinine in the 2^nd^ or 3^rd^ trimester, 2) treatment with SP (for case-management) in the 2^nd^ or 3^rd^ trimester, 3) treatment with ‘other antimalarials’ (any non-artemisinin) in the 2^nd^ or 3^rd^ trimester, 4) women who received IPT-SP for prevention of malaria, and 5) pregnancies which did not receive an antimalarial drug in the 2^nd^ or 3^rd^ trimester (i.e. because they did not have clinical malaria).

### Outcome definitions

Miscarriage was defined as pregnancy loss of <28 weeks of gestation and stillbirth as pregnancy loss of ≥28 weeks. This cut-off was chosen because the majority of the studies were conducted in resource-limited settings without access to neonatal intensive care. Fetal loss was defined as miscarriage or stillbirth. Studies that reported miscarriages but did not report the number of exposures occurring before 28 weeks of gestation or studies that reported miscarriages and stillbirths using different definitions, were included in the analyses of any fetal loss.

We defined congenital malformation as any major or minor anomaly. We included all reported congenital anomalies in the analysis because of inconsistencies in the definition of major congenital malformations. We grouped congenital anomalies by organ system according to classifications developed by the Antiretroviral Pregnancy Registry [21].

### Statistical Methods

Comparing risk estimates between exposure groups: To compare the risk between exposure groups, crude pooled odds ratios (PORs) and pooled risk differences (PRDs) were obtained from the reported count data. Our primary measure of association was a POR, but for analyses in which studies reported zero outcomes in both comparison arms, we conducted PRD models to allow all data to contribute to the model. We used a 0.5 continuity correction with a Mantel-Haenszel fixed effects model to correct for zero event cells [22]. As sensitivity analysis, we used random effects model with DerSimonian-Laird weighting and Peto Method with a 0.5 continuity correction, and Mantel-Haenzel fixed effects with continuity corrections of 0.01 and 0.69 [22].

As per the Cochrane Collaboration guidelines, I^2^ values >75% were considered indicative of considerable heterogeneity [23]. We conducted stratified analyses by study type, geographic location, and comparison drug exposures, as well as bias level where possible (i.e. low, moderate, high; S1 and S2 Tables).

## Results

**Fig 1:**
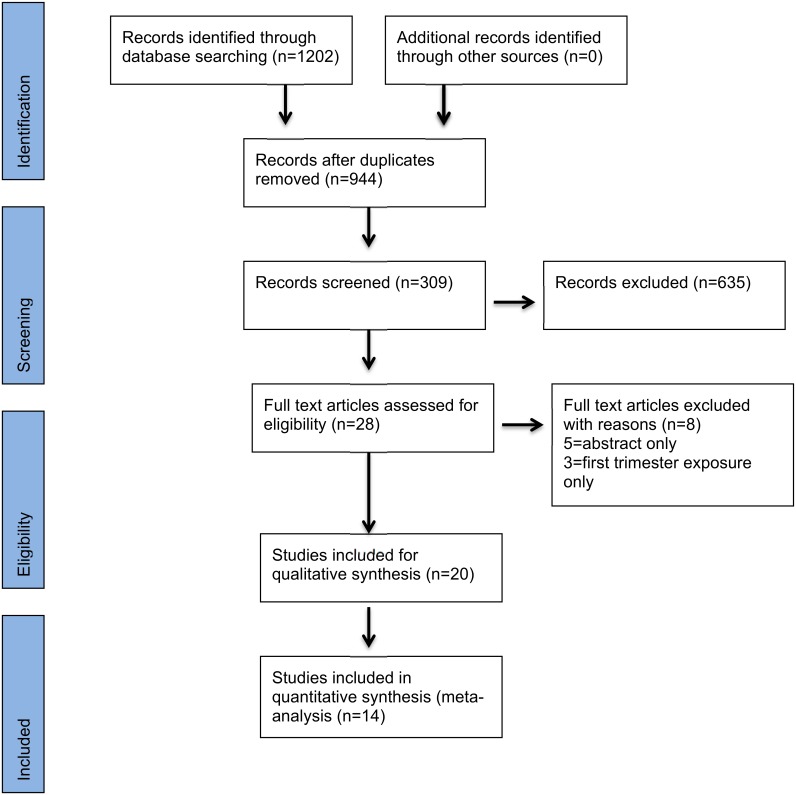
PRISMA flow diagram for search results June 15, 2015.

A total of 944 articles or reports were identified. After removal of duplications and review of titles, a total of 309 abstracts were reviewed and 20 studies (11 cohort studies and 9 RCTs, 10 from Africa and 10 from Asia) met the inclusion criteria and were included in at least one of the analyses (Fig 1). Six studies did not include comparisons with other non-artemisinins and were used only descriptively and not included in the pooled ORs, with the remaining 14 studies providing data for the pooled ORs. Twelve studies did not report if exposures occurred in the second or third trimester, and therefore were excluded from the analysis of miscarriage. The search did not identify any studies of artemisinins used for intermittent preventive treatment (IPT) of malaria during pregnancy that provided necessary data on pregnancy outcomes.

A total of 3,646 women in these 20 studies received an artemisinin during the 2^nd^ or 3^rd^ trimester of pregnancy (Tables 1 and 2). The most common exposures were: artemether-lumefantrine (AL) (N = 2,000), dihydroartemisinin piperaquine (DP) (N = 328), monotherapy with any artemisinin derivative including artemether and artesunate (N = 225), artesunate sulfadoxine-pyrimethamine (AS-SP) (N = 197), artesunate-mefloquine (AS-MQ) (N = 94), artesunate-amodiaquine (AS-AQ) (N = 83), and artesunate atovaquone-proguanil AAP (N = 39). One study reported 412 women receiving artesunate or artemether alone or in combination with MQ, clindamycin, atovaquone-proquanil (AP), artesunate iv, or AL but specific numbers were not reported by drug due to multiple treatments per patient [12]. There were 1,951 women who received other antimalarials in the 2^nd^ or 3^rd^ trimester including: chlorproguanil-dapsone (CD) (N = 81); chloroquine-SP (N = 24); mefloquine monotherapy (N = 36); quinine (N = 661); SP (for case-management) (N = 456); SP-AQ (N = 80), and SP-azithromycin (AZM) (N = 47), and for prevention IPTp-SP (N = 566). In these 20 studies, an additional 13,714 pregnant women reported no exposure to antimalarials and no recorded illness of malaria. Two studies with a no antimalarial comparison group were conducted in regions in sub-Saharan Africa where the risk of malaria is high [7,24]. The majority (n = 8194) of the women who were not exposed to an antimalarial were from one site in Thailand with a low risk of malaria where all patients were screened weekly for malaria, and therefore no IPTp-SP was provided [12].

**Table 1:** Description of cohort studies identified in the systematic review and included in the meta-analysis.

Study	Location and time period	Study Population	ACT Exposures	Comparator Exposures	2-3rd Trimester Outcomes
Adam, 2004 [25]	Sudan October 1997-February 2001	Pregnant women who presented with symptoms of P. falciparum malaria and had confirmed malaria parasites who were treated with quinine and returned to the hospital with recurrent malaria symptoms and parasite detected within three weeks. Mean age 27.1 years	Art im = 28 1st trim = 1/28 2nd trim = 12/28 3rd trim = 15/28	N/A	**Miscarriage = 0/12** **Stillbirth = 0/27** **CA = 0/27**
Adam, 2006 [26]	Sudan September 2004-March 2005	Pregnant women with uncomplicated P. falciparum malaria. Mean age 29.4 (s.d. 4.3) years	AS+SP = 32 2nd trim = 10/32 3rd trim = 22/32	N/A	**Miscarriage = 0/10** **Stillbirth = 0/32** **CA = 0/32**
Deen, 2001 [24]	Gambia March, 2000	All women of reproductive age (15–44 years) residing in the 42 study villages. Villages were part of a mass drug administration campaign. Pregnant women exposed to Mass drug administration were followed.	AS+SP = 287 1st trim = 77/287 2nd trim = 90/287 3rd trim = 28/287	Placebo = 40 No exp. = 132	**Miscarriage:** AS-SP = 0/90 Comm. +Placebo = 2/132 **Stillbirth:** AS-SP = 11/287 Comm. +Placebo = 6/172 **CA:** AS+SP = 6/287 Comm. +Placebo = 2/172
Manyando, 2010 [8]	Zambia October 2004-July 2008	Pregnant women attending antenatal clinic who were grouped based on the drug used to treat their last episode of malaria. Mean age NR.	AL = 495 1st trim = 159 2nd trim = 169 3rd trim = 162	SP = 506 1st trim = 125 2nd trim = 205 3rd trim = 176	**Miscarriage:** AL = 0/169 SP = 5/205 **Stillbirth:** AL = 9/473 SP = 13/478 **CA:** AL = 29/449 SP = 18/444
McGready, 2001 [12]	Thailand 1986–2001	Pregnant women who had microscopy confirmed P. falciparum or mixed P. falciparum and P. vivax infections. Mean age 24.8 (s.d. 6.4) years	Artesunate or artemether alone or in combination with MQ, C AP, or artesunate iv, or AL N = 461 1st trim = 40 2nd trim = 201 3rd trim = 211	No exp. = 8154	**Miscarriage:** Artemisinin = 20/201 Comm. = 1003/8154 **Stillbirth:** Artemisinin = 7/386 Comm. = 114/7058 **CA:** Artemisinin = 3/386 Comm. = 56/3707
*McGready, 1999 [27]	Thailand 1991–1996	Pregnant women in camps for refugees with uncomplicated, multi-drug resistant P. falciparum malaria.	AS = 61 2-3rd trim = 61 *study reports outcomes for 78 exposures	Q = 72 2-3rd trim = 72 MQ = 36 2-3rd trim = 36	**Stillbirth:** AS = 2/78 MQ or Q = 8/322 **Any Fetal Loss:** AS = 3/78 Q+MQ = 16/322 **CA:** AS = 0/73 MQ or Q = 4/322
Mosha, 2014[28]	Tanzania April-September 2012	2-3rd trimester pregnant and non-pregnant women with diagnosed uncomplicated malaria. P. falciparum detected by microscopy and hemoglobin level >/ = 7g/dl. Median age 25, range 18–41 years	AL = 55 2nd trim = 17 3rd trim = 16 Not pregnant AL N = 22	N/A	**Miscarriage: 0/17** **Stillbirth: 0/33** **CA: 0/33**
Rulisa, 2012[7]	Rwanda June 2007-July 2009	Pregnant women age 18+ treated with AL after diagnosis of simple P. falciparum malaria based on blood smear or clinical symptoms. Controls were pregnant women with no malaria. Age range 16–48.	AL = 1072 2nd trim = 434 3rd trim = 542	No exp. = 978	**Miscarriage:** AL 14/434 No antimalarial = 4/978 **Stillbirth:** AL = 31/1072 No antimalarial = 23/978 **CA:** AL = 3/1072 No antimalarial-3/978
Poespoprodjo, 2014 [29]	Indonesia April 2004-June 2009	All pregnant women and newborn infants admitted to maternity ward screened for malaria.	DP = 336 1st trim = 8 2-3rd trim = 328 DP+ivArt = 77 1st trim = 10 2-3rd trim = 67	Quinine = 347 iv Q 1st trim = 50 2-3rd trim = 259 CQ+SP = 24 2-3rd trim = 24 No exp. = 4408	**Stillbirth:** DP = 9/328 DP+ivART = 2/67 CQ+SP = 0/24 Q = 16/259 Community = 134/4408
Wang, 1989 [30]	China 1976–1980	Pregnant women with malaria with typical symptoms and signs; Plasmodium found in thick blood smear with a density over 500 mm3 and antimalarial had not been administered or with a known grade III chloroquine resistance. Mean age 25.8 (s.d. 4.1) years	Artemisinin in oil = 2 2nd trim = 2 Artemether = 4 2nd trim = 4	N/A	**Miscarriage:** Art oil = 0/2 Art = 0/4 **Stillbirth:** Art oil = 0/2 Art = 0/4 **CA:** Art oil = 0/2 Art = 0/4
^Nakelembe, 2012 [31]	Uganda and Burkina Faso	HIV negative women in the 2nd or 3rd trimester who were screened for malaria during normal IPT schedule and given AL if positive or SP if negative and followed through pregnancy	AL = 287 2-3rd trim = 287	IPT with SP = 566 2-3rd trim = 566	**Stillbirth:** AL = 3/287 IPT SP only = 7/566

*Pregnancy outcomes were reported for 78 artemisinin exposures, and 322 combined MQ and Q exposures. These ACT exposures are also included in the McGready 2001 cohort study with different comparison group (no antimalarial drug exposures).

^ Data provided by the authors. Trim trimester; ART im: artemether intramuscular; AS: artesunate; SP: sulfadoxine pyrimethamine; AL: artemether lumefantrine; CA: congenital anomal; MQ: mefloquine; C: Clindamycin; CD: chlorproguanal-dapsone; iv: intravenous; AP: atovaquone proquanil; Q: quinine; AS7: artesunate 7 days; CQ: chloroquine; AQ: amodiaquine; DP: dihydroartemisinin-piperaquine; IPT: intermittent preventative therapy; MDA: Mass drug administration; ANC: antenatal care; No exp: no exposure to antimalarials for treatment.

**Table 2:** Description of RCT studies identified in the systematic review and included in the meta-analysis.

Study	Location and time period	Study Population	ACT Exposures	Comparison Exposures	2-3rd Trimester Outcomes
Bounyasong, 2001 [32]	Thailand January 1995-December 1998	Pregnant women with P. falciparum, not more than 4% parasitized red cells, gestational age at least 28 weeks estimated by ultrasound.	AS+MQ = 28 2-3rd trim = 28	Q = 29 2-3rd trim = 29	**Any Fetal Loss:** AS-MQ = 0/28 Q = 0/29 **CA** AS-MQ = 0/28 Q = 0/29
Kalilani, 2007 [33]	Malawi September 2003-September 2004	Pregnant women (EGA 14–26 weeks) between 15 and 49 years old, with peripheral P. falciparum parasitemia; method of measuring gestational age not described. Median age 20 (range 17–24).	AS+SP = 47 2-3rd trim = 47/47	SP = 47 2-3rd trim = 47 SP+AZM = 47 2-3rd trim = 47	**Stillbirth:** AS-SP = 4/38 SP = 1/38 SP+AZM = 0/42 **Any fetal loss:** AS-SP = 4/38 SP = 0/38 SP+AZM = 4/42 **CA:** AS-SP = 0/38 SP = 0/38 SP-AZM = 0/42
McGready, 2000 [11]	Thailand October 1995-July 1997	Pregnant women in 2nd or 3rd trimester, estimated by fundal height seen at ANC who had microscopy confirmed uncomplicated P. falciparum infection	AS-MQ = 66 2-3rd trim = 66	Q = 42 2-3rd trim = 42	**Any Fetal Loss:** AS-MQ = 2/66 Q = 0/42 **CA:** AS-MQ = 0/66 Q = 0/42
McGready, 2001 [34]	Thailand October 1997-January 2000	Pregnant women in 2nd or 3rd trimester estimated by fundal height seen at ANCs who had microscopy confirmed P. falciparum infections. Age range 15–41 years	AS7 N = 64 2-3rd trim = 64	Q+C N = 65 2-3rd trim = 65	**Stillbirth:** AS7 = 1/64 Q+C = 1/65 **Any Fetal Loss:** Art = 1/64 Q+C = 1/65 **CA:** Art = 0/64 Q+C = 1/65
McGready, 2005 [35]	Thailand December 2001-July 2003	Pregnant women with first episode of P. falciparum or mixed infection (P. vivax), 14–31 weeks gestation estimated by ultrasound or fundal height, Ht > = 20%. Mean age 26 (s.d. 7) years	AAP N = 39 2-3rd trim = 39	Q N = 42 2-3rd trim = 42	**Any Fetal Loss:** AAP = 0/34 Q = 0/38 **CA:** AAP = 2/34 Q = 1/38
McGready, 2008 [10]	Thailand April 2005-August 2006	Patients with acute P. falciparum malaria in 2nd or 3rd trimester estimated by ultrasound. Originally only allowed 2nd infection in pregnancy (already failed quinine), but later widened to allow first infections in pregnancy. Age range 14–44 Years	AL N = 125 2-3rd trim = 125 AS7 N = 128 2-3rd trim = 128		**Stillbirth:** AL = 1/117 AS7 = 1/120 **Any Fetal Loss:** AL = 1/117 AS7 = 2/120 **CA:** AL = 3/117 AS7 = 4/120
Mutabingwa, 2009 [36]	Tanzania January 2004-September 2006	Pregnancy with either a positive blood smear for P. falciparum with at least 800 parasites/μL in an asymptomatic woman or any of the following symptoms within 2 days prior to consultation: history of fever, headache, vomiting, chills/rigors and/or any of the following signs: temperature ≥37.5 & <39.5°C, Hb ≥7 and <9 g/dl) with P. falciparum parasitemia at any density. All cases were between 14–34 weeks gestation defined by presence of fetal heartbeat. Median age 21 years	AS-AQ = 83 2-3rd trim = 83	SP = 28 2-3rd trim = 28 SP-AQ = 80 2-3rd trim = 80 CD = 81 2-3rd trim = 81	**Any Fetal Loss:** AS-AQ = 4/83 SP-AQ = 1/80 SP = 0/28 CD = 1/81 CA:* AS+AQ = 3/83 SP = 3/28 CD = 7/81 SP+AQ = 8/80
Piola, 2010 [6]	Uganda October 2006-May 2009	Women with viable pregnancy with an estimated gestation of ≥13 weeks determined by ultrasound or LMP and malaria infection detected by microscopy (P. falciparum mixed or monoinfection). SP may have been used for prevention before entry to the study and some inadvertently when entered.	AL = 152 2-3rd trim = 152	Q = 152 2-3rdtrim = 152	**Stillbirth:** AL = 2/144 Q = 3/137 **Any fetal loss:** AL = 5/144 Q = 7/137 **CA:** AL = 3/144 Q = 2/137
Sowunmi, 1998 [37]	Nigeria January 1994-March 1997	All patients referred to University College Hospital with persistent P. falciparum parasitemia and acute uncomplicated malaria after failure of supervised therapy with standard regimen of chloroquine or after a single dose of SP or both CQ and SP. Oral fluid intolerance, no history of allergy to known antimalarial drugs, 2nd or 3rd trimester determined by ultrasound	Art+MQ = 23 2-3rd trim = 23 Art im = 22 2-3rd trim = 22		**Stillbirth:** Art+MQ = 0/23 Art im = 0/22 **Any fetal loss:** Art+MQ = 0/23 Art im = 0/22 **CA:** Art+MQ = 0/23 Art im = 0/22

*Only minor CA. Trim: trimester; ART im: artemether intramuscular; AS: artesunate; AZM: azithromycin; SP: sulfadoxine pyrimethamine; AL: artemether lumefantrine; CA: congenital anomaly; C: Clindamycin; MQ: mefloquine; CD: chlorproguanal-dapsone; iv: intravenous; AP: atovaquone proquanil; Q: quinine; AS7: artesunate 7 days; CQ: chloroquine; AQ: amodiaquine; DP: dihydroartemisinin-piperaquine; IPT: intermittent preventative therapy.

Ten studies reported data on presence of fever at enrollment, and 12 studies reported enrollment parasitemia (S3 Table). The mean gestational age at enrollment ranged from 21 weeks to 38 weeks across the studies. Only three studies reported data on HIV status, preventing stratification by HIV status [8,33,36]. Further details about the characteristics of included women can be found in S3 Table.

### Miscarriage

Eight studies reported data on late miscarriages after receipt of an artemisinin in the second trimester, but only four studies reported comparisons with pregnant women who did not receive artemisinins and there were no studies with a quinine arm. A total of 34 miscarriages were reported among 939 women given artemisinins, including data from 5 studies reporting no miscarriages among 304 exposures.

#### Comparison to SP for treatment

Only one study compared use of artemisinins to use of SP, with zero miscarriages among 169 ACTs exposures and 5 miscarriages among 205 SP exposures.

#### Comparison to no antimalarial drug exposure

**Fig 2:**
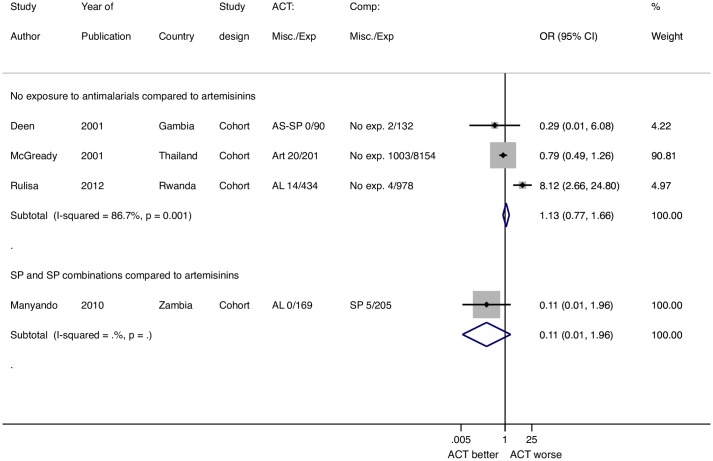
Pooled odds ratio for miscarriage after 2nd trimester exposures to artemisinins stratified by comparison group. *McGready 2001 reported multiple types of artemisinin exposures that were combined for this analysis (12). ART artemisinins, AL artemether-lumefantrine, AS-SP artesunate sulfadoxine pyrimethamine, SP sulfadoxine pyrimethamine, No exp. No exposure to antimalarials.

Three studies compared artemisinin users to women who did not receive an antimalarial drug. There were 34 miscarriages among 725 women who received an artemisinin, and 1009 miscarriages among 9264 women who received no antimalarials and therefore were unlikely to have malaria (POR = 1.13, 95% CI 0.77–1.66, I^2^ = 86.7%, 3 studies) (Fig 2).

### Stillbirth

Sixteen studies reported a total of 83 stillbirths among 3,595 women who received an artemisinin in the 2nd or 3rd trimester.

#### Comparison to Quinine

**Fig 3:**
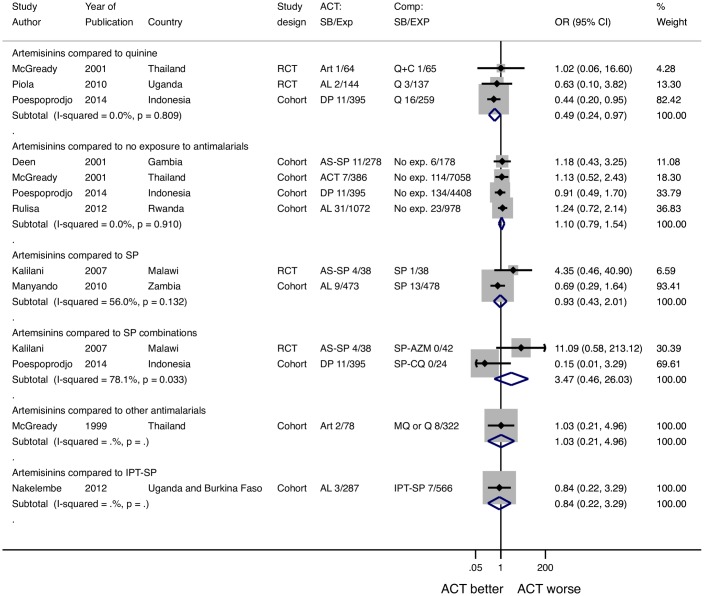
Pooled odds ratio for stillbirth after 2-3rd trimester exposures to artemisinins compared to other drugs. *McGready 2001 reported multiple types of artemisinin exposures that were combined for this analysis and reported as ACT (12). ^McGready 1999 MQ or Q exposures include patients given MQ, Q, or both (33). ART: artesunate, AL: artemether-lumefantrine, AAP: artesunate atovaquone proguanil, AS-AQ: artesunate-amodiaquine, Q: quinine, Q+C: quinine+clindamycin, SP: sulfadoxine pyrimethamine, AQ: amodiaquine, SP+CQ: sulfadoxine pyrimethamine chloroquine, MQ: mefloquine, SP-AZM: sulfadoxine pyrimethamine azithromycin, CD: chlorproguanal-dapsone, IPT: intermittent preventative treatment, RCT: randomized controlled trial, ACT: artemisinin combination therapy, No exp. no exposure to antimalarials.

In studies with a quinine comparison arm, there were 14 stillbirths among 603 women who received an artemisinin and 20 stillbirths among 461 women who received quinine (POR 0.49, 95% CI 0.24–0.97, I^2^ = 0%, 3 studies) (Fig 3).

#### Comparison to SP treatment

In the two studies comparing use of artemisinins to SP for case-management, there were 13 stillbirths among 511 women who received an artemisinin, and 14 stillbirths among 516 women who received SP (POR 0.93, 95% CI 0.43–2.01, I^2^ = 56%, 2 studies).

#### Comparison to other antimalarials for treatment

Receipt of an artemisinin was not associated with a clear increase in the risk of stillbirth compared to receipt of SP combinations (POR 3.47, 95% CI 0.46–26.03 I^2^ = 100%, 2 studies); these results were based on only two studies with considerable evidence of heterogeneity. Receipt of artemisinin compared to mefloquine and/or quinine for the treatment of malaria was not associated with an increased risk of stillbirth OR 1.03 (95% CI 0.21–4.96).

#### Comparison to IPT-SP

Only one study compared women who received artemisinins for treatment to women who only received IPT-SP. There was no increased risk of stillbirth after receipt of an artemisinin compared to women who received only IPT-SP, OR = 0.84 (95% CI 0.22–3.29).

#### Comparison to no antimalarial drug exposure

In the studies that compared use of an artemisinin to no exposure to antimalarials, there were 60 stillbirths among 2131 women who received an artemisinin, and 277 among 12,622 women who received no antimalarials (POR 1.10, 95% CI 0.79–1.54, I^2^ = 0%, 4 studies).

### Any Fetal Loss

In twelve studies that did not specify if exposures occurred in the 2^nd^ or 3^rd^ trimester, there were 38 fetal losses among 1505 women who received an artemisinin in the 2 or 3^rd^ trimester.

#### Comparison to Quinine

**Fig 4:**
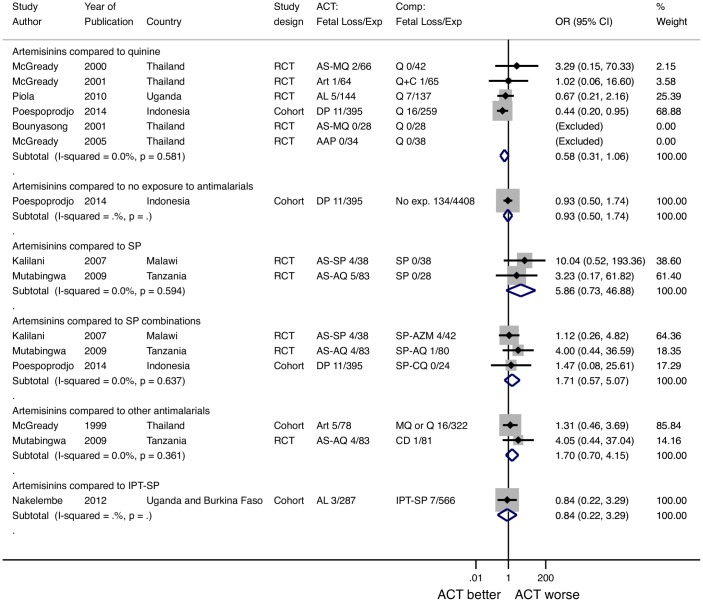
Pooled Odds ratio for fetal loss after 2-3rd trimester exposures to artemisinins stratified by comparison group. *McGready 2001 reported multiple types of artemisinin exposures that were combined for this analysis and reported as ACTs (12). ^McGready 1999 MQ or Q exposures include patients given MQ, Q, or both (33). ART: artesunate, AS-MQ: artesunate mefloquine, AL: artemether-lumefantrine, DP: dihydroartemisinin-piperaquine, AAP: artesunate atovaquone proguanil, AS-SP: artesunate sulfadoxine pyrimethamine, AS-AQ: artesunate-amodiaquine, Q: quinine, Q+C: quinine+clindamycin, SP: sulfadoxine pyrimethamine, AQ: amodiaquine, SP+CQ: sulfadoxine pyrimethamine chloroquine, MQ: mefloquine, SP-AZM: sulfadoxine pyrimethamine azithromycin, CD: chlorproguanal-dapsone, IPT: intermittent preventative treatment, RCT: randomized controlled trial, ACT: artemisinin combination therapy, No exp. No exposure to antimalarials.

In the 6 studies with a quinine arm, there were 19 fetal loses among 731 women who received artemisinins, and there were 24 fetal losses among 559 quinine exposures (POR = 0.58, 95% CI 0.31–1.06, I^2^ = 0%, 6 studies) (Fig 4).

#### Comparison to SP for treatment

In the two studies with an SP arm, there were 9 fetal losses among 121 women who received an artemisinin and there were zero fetal losses among 66 women who received SP for treatment. (POR 5.86, 95% CI 0.73–46.88, I^2^ = 0%, 2 studies).

#### Comparison to other non-artemisinin antimalarials for treatment

Receipt of artemisinins in the 2^nd^ or 3^rd^ trimester was not associated with an increased risk of fetal loss compared to SP combinations or other antimalarials (POR 1.71, 95% CI 0.57–5.07, I^2^ = 0%, 3 studies, and POR 1.70 95% CI 0.70–4.15, I^2^ = 0%, 2 studies, respectively).

#### Comparison to IPT-SP

Only one study compared artemisinin to IPT-SP. There was no increased risk of fetal loss among women who received an artemisinin for treatment compared to women who only received IPT-SP (OR = 0.84, 95% CI 0.22–3.29).

#### Comparison to no antimalarial drug exposure

Based on the results of a single study, there was no increased risk of fetal loss among women who received an artemisinin compared to women who received no antimalarial drugs (OR = 0.93, 95% CI 0.50–1.74).

### Congenital Anomalies

Thirteen studies reported a total of 55 congenital anomalies among 2,807 exposures to artemisinins in the 2nd or 3^rd^ trimesters.

#### Comparison to quinine

**Fig 5:**
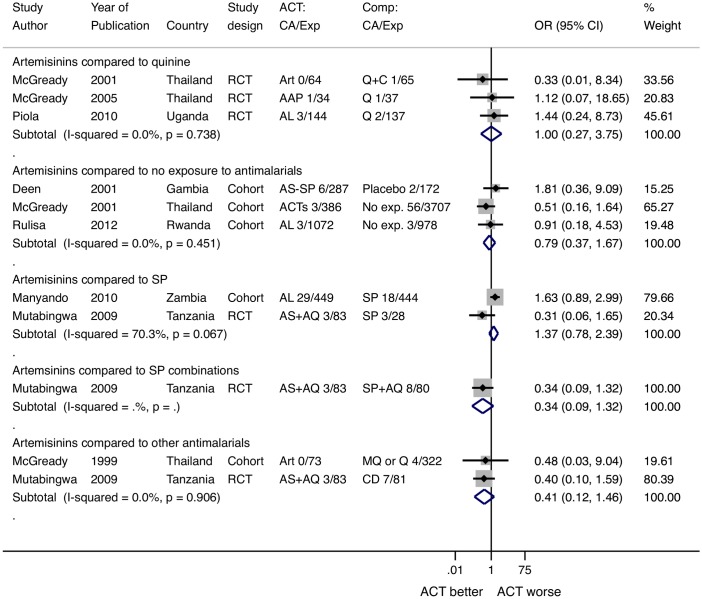
Pooled odds ratio for congenital anomalies after 2-3rd trimester exposures to artemisinins stratified by comparison group. *McGready 2001 reported multiple types of artemisinin exposures that were combined for this analysis and reported as ACTs (12). ART: artesunate, AL: artemether-lumefantrine, AAP: artesunate- atovaquone proguanil, AS-AQ: artesunate-amodiaquine, Q: quinine, Q+C: quinine+clindamycin, SP: sulfadoxine pyrimethamine, AQ: amodiaquine, SP+AQ: sulfadoxine pyrimethamine amodiaquine, MQ: mefloquine, CD: chlorproguanal-dapsone, RCT: randomized controlled trial, ACT: artemisinin combination therapy, No exp. No exposure to antimalarials.

There were 4 congenital anomalies among infants born to 242 women who received an artemisinin and 4 congenital anomalies among 239 women who received quinine (POR = 1.00, 95% CI 0.27–3.75, I^2^ = 0%, 3 studies) (Fig 5).

#### Comparison to SP for treatment

In the two studies comparing artemisinins to SP, there were 32 congenital anomalies among 532 women who received an artemisinin and 21 congenital anomalies among 472 women who received SP (POR = 1.37, 95% CI 0.78–2.39, I^2^ = 70.3%, 2 studies).

#### Comparison to no antimalarial drug exposure

In studies with women who were not exposed to antimalarials in pregnancy, there were 12 congenital anomalies among 1745 women who received an artemisinin and 64 congenital anomalies among 4857 women who did not receive antimalarials in pregnancy (POR 0.79, 95% CI 0.37–1.67, I^2^ = 0%, 3 studies).

Across the 13 studies reporting data on congenital anomalies, the most common congenital anomaly reported was umbilical hernia; 21 among infants exposed in utero to ACT during the 2 or 3^rd^ trimester and an additional 12 umbilical hernias reported among 1,113 infants whose mother used other antimalarials during pregnancy. There were three central nervous system (CNS) defects among infants exposed to ACTs (anencephaly, hydrocephaly, and hemimegaloencephaly), but no CNS defects were reported among infants exposed to other antimalarials. One heart defect was reported among artemisinin exposed infants (acyanotic heart disease), and none among infants exposed to other antimalarials or no antimalarials in the 2 or 3^rd^ trimester of pregnancy (Table 3). Studies did not routinely report congenital anomalies stratified by major and minor anomalies; therefore, it was not possible to conduct an analyses of the risk of any major anomaly. Furthermore, small numbers of events precluded the analysis of congenital anomalies stratified by organ system.

**Table 3:** Reported congenital anomalies grouped by organ system and stratified by antimalarial drug exposure during 2-3^rd^ trimester of pregnancy.

Group and Malformation*	No antimalarial exposure	ACT	Other antimalarial
Central Nervous System (CNS)			
Anencephaly		1	
Spina bifida	1		
Hydrocephalus		1	
Unspecified neurologic disorder	1		
Hemimegaloencephaly		1	
Face and Neck (FACE)			
Midline cyst on nose§		1	1
Unspecified anomaly of ear			1
Unspecified anomaly of eye		1	
Nose-small§		1	
Aglossia		1	
Obstructive Heart Defects—Left Sided (CV-LT)			
Acyanotic heart disease		1	
Respiratory System (RES)			
Thoracic asymmetry	1		
Female Genitalia (G-FEMALE)			
Small labia§		1	
Male Genitalia (G-MALE)			
Undescended testicles		2	
Hernia inguinal			1
Limb Reduction/Addition Defects (LIMB)			
Other and unspecified polydactyly		5	6
Hyperextensibility of joint§		1	
Specified or unspecified reduction defect of unspecified limb		1	
Other Musculoskeletal Defects (MS-O)			
Umbilical hernia§		21	12
Human tail		1	
Unspecified malformation of left foot		1	
Skin and Skin Derivatives (SKIN)			
Jaundice§	1		
Hyperpigmentation§		1	
Dermal Cyst§			1
Lanugo§		1	
Haemangioma	1		
Unspecified anomaly of skin		2	
Chromosome Anomaly (CHROM)			
Trisomy 18		1	
Trisomy 21		1	
Other Organs and Organ Systems (OTHER)			
Amniotic banding		1	
Alagille's (genetic)		1	
Unspecified anomaly	56	6	23
Total malformations	61	55	45

*No cleft lip and/or palate, conotruncal heart defects, obstructive heart defects—right sided, other heart defects, other circulatory system, upper gastrointestinal system, lower gastrointestinal system, or renal and urinary system defects were reported.

^§^Denotes reported congenital anomalies that do not meet the inclusion criteria for the Antiretroviral Pregnancy Registry’s Organ Classification System.

### Sensitivity Analyses

In order to assess the robustness of our findings for our POR models we conducted sensitivity analyses using different correction factors for zero cells (0.69 and 0.01), and two different pooling techniques, Peto method and random effects models, given that there is a lack of consensus in the literature on the most appropriate method for modeling rare outcomes [22]. Sensitivity analyses using different correction factors for zero cells (0.69 and 0.01), and two different pooling techniques, Peto method and random effects models, did not change the overall interpretation of the analyses, but the results were most sensitive to changes in the correction factor and not the modeling technique (S4 Table).

## Discussion

There was no indication that the risk of late miscarriage was higher in artemisinin recipients compared to women who received no antimalarials in the 2^nd^ trimester or to SP for treatment, although the latter comparison included only a single study. There were fewer studies that compared the risk of late miscarriage, but in 3 studies that compared the risk against women who received no antimalarials in the 2^nd^ trimester, and in 1 study against women who received SP for treatment, there was no evidence for an increased risk. The risk of stillbirths in women treated with artemisinins in the 2^nd^ or 3^rd^ trimester was lower compared to those treated with oral quinine during the same gestational period. Additionally, there was no increased risk of stillbirth after receipt of an artemisinin compared to receiving other antimalarials for treatment, IPT-SP only, or receiving no antimalarials. No increased risk was observed for fetal loss or congenital abnormalities when compared to quinine, SP, other antimalarials, IPT-SP, or to women who did not receive any antimalarials.

This meta-analysis of over 3500 artemisinin exposures builds upon the existing literature by including monotherapy and multiple artemisinins combinations therapies and using multiple comparison groups. Previous WHO guidelines in 2002 and 2006 were developed based on the findings for 607 and 1000 2^nd^ or 3^rd^ trimester exposure respectively [38,39]. Specifically, the results support the findings of a previous meta-analyses of the safety of the artemether-lumefantrine (AL) used in pregnancy which showed that AL was not associated with an increased risk of adverse pregnancy outcomes compared to quinine and SP for treatment [40]. Additionally these results complement recent findings from an updated analysis of first trimester malaria on the Thailand/Burma border which found no difference in the risk of miscarriage between women treated with an artemisinin compared to women treated with quinine [41].

This study has several limitations. The findings from the 11 cohort studies may be confounded by indication due to the non-random administration of treatment. Of the nine studies that randomized patients to a treatment, five compared an artemisinin to quinine, so confounding is unlikely to be an important concern for this comparison. Confounding due to malaria infection is of particular concern in the studies comparing ACTs for treatment to no antimalarials, because these studies did not take into account the effect of malaria on poor pregnancy outcomes. Despite this limitation, we found equivalent risk of adverse pregnancy outcomes for women who received artemisinins compared to women who received other therapies and no antimalarials. Additionally because of small sample sizes, we were unable to stratify our analyses by potential effect modifiers such as geography (Asia vs. Africa), which may account for the observed heterogeneity of our results given the different epidemiology of malaria in the two regions. Furthermore, only 3 studies reported any data on HIV infection. Included studies had limited ability to detect an effect on miscarriages because of their recruitment strategies: recruiting women from ANCs can lead to underestimates of the occurrence of miscarriage because women often present late in the 2nd trimester, and women who have an early miscarriage will not present at all, limiting the interpretation of the findings. Additionally, there may be further misclassification of outcome due to limitations of the gestational age assessment. Finally, there may be heterogeneity in the rigor by which studies assessed outcomes such as congenital anomalies. Although each study contributing to the analysis was assessed for bias using either the Cochrane Collaboration Tool or the Newcastle Ottawa Scale, these tools may not fully capture all sources of bias in the assessment of safety outcomes.

This analysis was limited to artemisinin use for case-management only. Recently two prevention studies which randomized pregnant women to IPT with DP were published [42,43]. As more studies of ACT used for IPT with pregnancy outcomes become available, additional meta-analyses of safety outcomes may be warranted, especially if they enroll women from first-trimester.

Despite identifying over 3500 women who received artemisinins in the 2^nd^ or 3^rd^ trimester, the sample size was too limited to allow the evaluation of the risks of specific congenital anomalies. Also, in most studies the ascertainment of anomalies was based only on surface exams conducted at birth which would not detect congenital anomalies of internal organs or congenital anomalies of the brain leading to functional deficits. According to the Centers for Disease Control and Prevention, major congenital anomalies in the U.S. are detected in 5% of children by age 5, but only 60% of these anomalies are detected at birth [44]. The studies included in the analyses reported congenital anomalies (major and minor) among 1% of births or less. This low proportion is likely due to the fact that birth defects such as cardiac defects and those related to brain development would not be detected by surface exams, and therefore this study cannot rule out or estimate the risk of these specific anomalies. Given that the brain is still developing through the 2^nd^ and 3^rd^ trimester, it would be important to assess exposed infants later in infancy or childhood to rule out an increased risk of these types of defects. In the review, only three studies followed infants up to one year after birth [10,11,35].

The results of this study build upon the limited data to-date on the safety of ACT used in pregnancy. These data suggest that the risk of miscarriage and congenital anomalies is similar among women treated with artemisinins in 2^nd^ or 3^rd^ trimester of pregnancy and women treated with quinine or other non-artemisinin antimalarials. Furthermore, the risk of stillbirth was lower compared to quinine recipients, possibly reflecting a higher efficacy of artemisinins against infection [45]. Though these results were based on few exposures across a limited number of studies, they support the current WHO guidelines recommending the use of ACTs for treatment of malaria in the 2nd or 3rd trimester of pregnancy.

## Supporting Information

S1 Fig PICOTS Framework of the systematic search. (TIFF)

S2 Fig PubMed Search Strategy, Search Date June 15, 2015. (TIFF)

S3 Fig Pooled Risk Difference for fetal loss after 2-3^rd^ trimester exposures to artemisinins, stratified by comparison group. (TIFF)

S1 PRISMA Checklist (DOC)

S1 Protocol (DOCX)

S1 Supplementary Methods (DOCX)

S1 Table Newcastle Ottawa Scale Assessing Bias in Cohort Studies. (DOCX)

S2 Table Cochrane Bias Assessment of Randomized Controlled Trials. (DOCX)

S3 Table Clinical presentation of pregnant women upon enrollment from included studies. (DOCX)

S4 Table Sensitivity analyses of pooled odds ratios using different modeling techniques and correction factors. (DOCX)
