# Supplementary figures and images for: The Safety of Artemisinin Derivatives for the Treatment of Malaria in the 2nd or 3rd Trimester of Pregnancy: A Systematic Review and Meta-Analysis
Source: PLoS One. 2016 Nov 8;11(11):e0164963. doi: 10.1371/journal.pone.0164963 (PMC5100961; doi:10.1371/journal.pone.0164963)

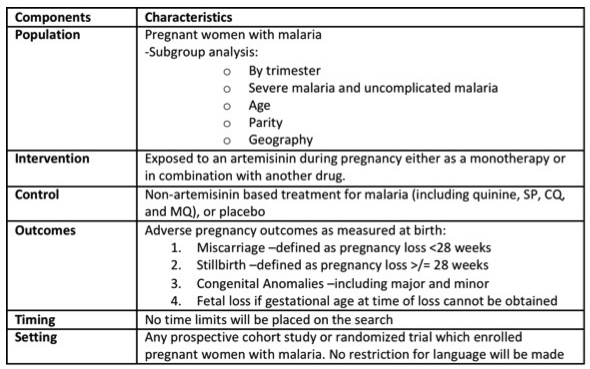

Supplement: S1 Fig — (TIFF) [file pone.0164963.s001.tiff]

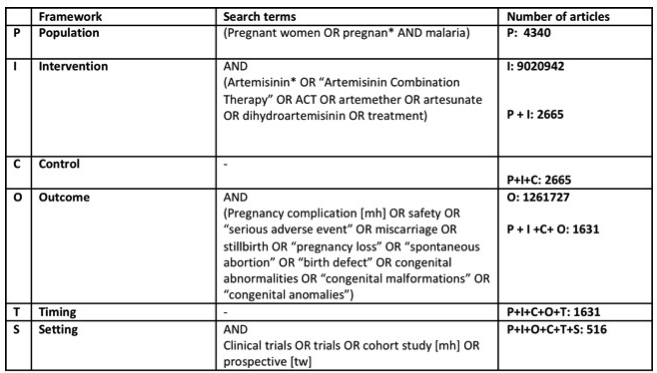

Supplement: S2 Fig — (TIFF) [file pone.0164963.s002.tiff]

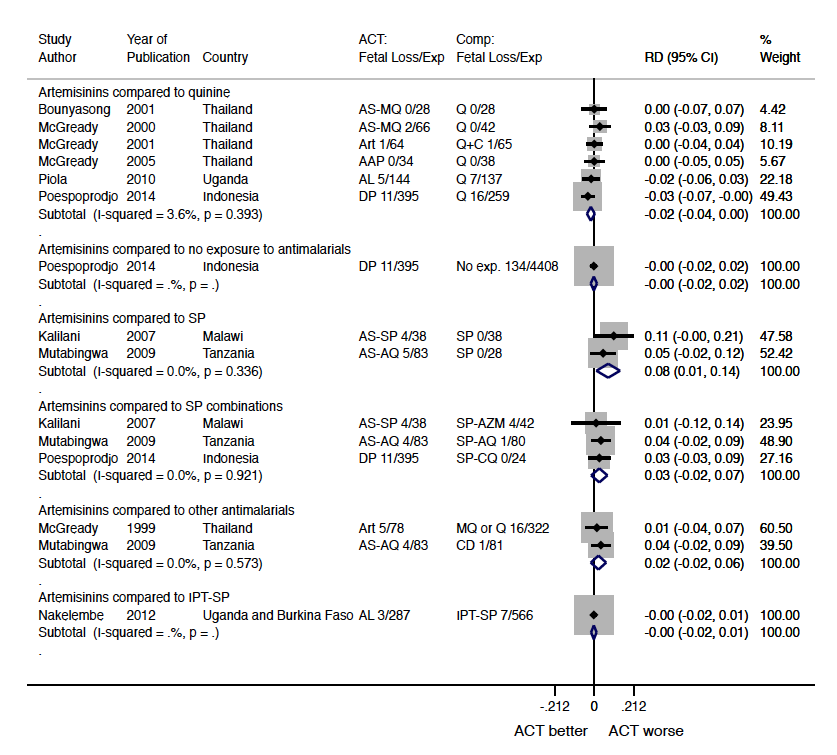

Supplement: S3 Fig — (TIFF) [file pone.0164963.s003.tiff]
