# Supplementary material for: The Safety of Artemisinin Derivatives for the Treatment of Malaria in the 2nd or 3rd Trimester of Pregnancy: A Systematic Review and Meta-Analysis
Source: PLoS One. 2016 Nov 8;11(11):e0164963. doi: 10.1371/journal.pone.0164963 (PMC5100961; doi:10.1371/journal.pone.0164963)
Supplement: S1 Protocol — (DOCX) [file pone.0164963.s005.docx]

**The Safety of artemisinins in pregnancy for the treatment of malaria: A systematic review and meta-analysis**

**Systematic Review Protocol**

Kovacs S.^1a^, van Eijk A.^2^, Stergachis A.^1,3^, ter Kuille F.^3,4^

1. Department of Epidemiology, University of Washington, Seattle, WA, USA
2. Liverpool School of Tropical Medicine, Liverpool, UK
3. Department of Global Health, University or Washington, Seattle, WA, USA
4. Kenya Medical Research Institute (KEMRI) Centre for Global Health, Kisumu, Kenya

^a^ Corresponding Author:

Stephanie Kovacs, MPH

School of Public Health, University of Washington

Health Sciences Building, F-342 Box 357236 Seattle, WA 98195

**Author Contributions:**

SK wrote the protocol with input from AVE, AS and FTK. SK developed the search terms. SK and AVE will review all abstracts independently, and AS will serve as the tiebreaker. SK and AVE will abstract all data independently. SK will conduct all analyses.

**Guarantor:**

Feiko ter Kuille on behalf of the Malaria in Pregnancy Consortium

**Support:**

This work is partially supported by the TL1 TR000422 from the NIH National Center for Advancing Translational Sciences. The supporter had no role in the development of the protocol.

**INTRODUCTION**

**Rationale:**

This systematic review addresses a key problem in malaria control efforts - the safety of artemisinins in pregnancy. In 2007, an estimated 54.7 million pregnancies occurred in areas of stable *P. falciparum* malaria transmission and an additional 70.5 million occurred in areas of low transmission or areas with only *P. vivax* malaria (1,2). Malaria is responsible for up to 100,000 neonatal deaths due to being born small for gestational age (SGA) and 10,000 maternal deaths (3,4). Severe malaria may lead to abortion, stillbirth, prematurity and low birth weight (4). In addition, malaria is associated with maternal anemia which can lead to death if severe (4). Given the high morbidity and mortality associated with malaria in pregnancy, safe and efficacious drugs are needed for treatment and prevention, but because of ethical concerns, few studies enroll pregnant women (5,6).

Studies of rats and rabbits have found artemisinins to be embryotoxic and teratogenic (7). A review of all antimalarial drugs used in pregnancy noted artesunate is very toxic to rat and rabbit embryos, with fetal reabsorption in rats reported at low doses (28-223 mg/kg/day) given orally on days 9-14 of gestation (5). High rates of congenital anomalies, including bent and/or shortened long bones and treatment related heart defects, were observed in rat litters (5). Primate studies demonstrated similar embryo toxicity, with 55% and 100% embryo lethality for 12 and 30 mg/kg/d oral doses respectively (8). In addition, observations of three live embryos from the 30mg group noted reduction in blood cells in the vasculature and the cardiac chambers were distended with thin walls (8). The primary embryonic targets for artemisinins are the primitive erythroblasts proliferating during gestational periods of 10-14 days in the rat and 18-40 days in the monkey. In humans this corresponds to a sensitive period from post conception day 21 to approximately post conception week 9 (8).

Despite the concerning data from animal studies limited human studies of artemisinins show no increased risk of adverse pregnancy outcomes including miscarriage, stillbirth or congenital anomalies. Five cohort studies and three randomized clinical trials have studied the safety of artemisinins in pregnancy (9–15) but all had small sample sizes and were not powered to examine safety outcomes. Of these eight studies, four reported exposures to artemisinins during the first trimester and four reported outcomes from the same cohort of women attending health centers in the refugee camps along the Thailand-Burma border. No study found a statistically significant increased rate of spontaneous abortions, stillbirths, neonatal deaths, prematurity, or congenital anomalies. The highest prevalence of congenital anomalies was observed in women exposed to AS during the first trimester (4.5%) but it was not statistically significantly higher than the prevalence of 1% observed in women not exposed to artemisinins during the first trimester (12). All eight studies were limited by small sample sizes to detect rare but clinically significant differences in the rates of adverse pregnancy studies. By pooling data from randomized controlled trials and prospective cohort studies through meta-analysis, we will increase the power and thoroughly assess the risk of adverse pregnancy outcomes associated with exposure to artemisinins during pregnancy.

**Research question:**

Are women exposed to artemisinins during pregnancy at increased risk of adverse pregnancy outcomes including miscarriage, stillbirth, and congenital anomalies compared to women exposed to quinine and other non-artemisinin based antimalarials?

**Primary Objectives:**

1. To estimate the pooled event rate for miscarriage, stillbirth, and congenital anomalies associated with exposure to artemisinins for treatment during pregnancy.
2. To estimate the pooled relative risk and risk difference for miscarriage, stillbirth, and congenital anomalies associated with exposure to artemisinins for treatment during pregnancy compared to women exposed to non-artemisinin based antimalarials.
3. To estimate the pooled relative risk and risk difference for miscarriage, stillbirth and congenital anomalies among women exposed to specific ACT combinations for treatment compared to each other (e.g. artesunate-mefloquine compared to artemether-lumefantrine) using direct and indirect comparisons from randomized controlled trials.

**METHODS**

**Eligibility Criteria:**

Due to a limited number of randomized controlled trials including pregnant women, we will include both prospective cohort studies and trials. Our search will include pregnant women of any age with malaria regardless of severity. We will stratify our analyses by age groups, malaria severity (uncomplicated and severe), and parity (<2 and 2+). For details of the eligibility criteria, see the PICOTS Framework.

**PICOTS Framework:**

| **Components** | **Characteristics** |
| --- | --- |
| **Population** | Pregnant women with malaria  -Subgroup analysis:   - - By trimester   - Severe malaria and uncomplicated malaria   - Age   - Parity   - Geography |
| **Intervention** | Exposed to an artemisinin during pregnancy either as a monotherapy or in combination with another drug. |
| **Control** | Non-artemisinin based treatment for malaria (including quinine, SP, CQ, and MQ), or placebo |
| **Outcomes** | Adverse pregnancy outcomes as measured at birth:   1. Miscarriage –defined as pregnancy loss <28 weeks 2. Stillbirth –defined as pregnancy loss >/= 28 weeks 3. Congenital Anomalies –including major and minor |
| **Timing** | No time limits will be placed on the search |
| **Setting** | Any prospective cohort study or randomized trial which enrolled pregnant women with malaria. No restriction for language will be made |

**Search Strategy:**

An electronic literature search applying the aforementioned PICOTS framework will be conducted using the following clinical databases: MEDLINE, EMBASE, and the Malaria in Pregnancy Consortium (MiPc) Library (16). A multi-concept Boolean search strategy will be applied using keywords and MeSH. We will additionally search 'gray literature' databases, conference abstracts, manually review reference lists of selected publications as well as records recommended by contacting experts so as to encompass a broad range of available literature. A librarian will be involved in this search and the strategy optimized for each database searched. We will merge citations from all individual databases into one citation software file. Duplicates will be removed and the last date of the search documented.

PubMed Search Strategy: Search Date January 12, 2015

|  | **Framework** | **Search terms** | **Number of articles** |
| --- | --- | --- | --- |
| **P** | **Population** | (Pregnant women OR pregnan* AND malaria) | **P:  4138** |
| **I** | **Intervention** | AND  (Artemisinin* OR “Artemisinin Combination Therapy” OR ACT OR artemether OR artesunate OR dihydroartemisinin OR treatment) | **I: 8616747**  **P + I: 2519** |
| **C** | **Control** | - | **C+P+I: 2519** |
| **O** | **Outcome** | AND  (Pregnancy complication [mh] OR safety OR “serious adverse event” OR miscarriage OR stillbirth OR “pregnancy loss” OR “spontaneous abortion” OR “birth defect” OR congenital abnormalities OR “congenital malformations” OR “congenital anomalies”) | **O: 1184241**  **P + I +C+ O: 1535** |
| **T** | **Timing** | - | **P+I+O+C+T: 1535** |
| **S** | **Setting** | AND  Clinical trials OR trials OR cohort study [mh] OR prospective [tw] | **P+I+O+C+T+S: 487** |

**EMBASE Search:** January 13, 2015: 295 Articles

'pregnant woman' AND malaria AND (artemisinin* OR 'artemisinin combination therapy' OR act OR artemether OR artesunate OR dihydroartemisinin) AND [embase]/lim NOT [medline]/lim AND 'human'/de

**Malaria in Pregnancy Library:** January 13, 2015: 384 Articles

Artemisinin OR artemether OR artesunate OR dihydroartemisinin

**Data Management:**

Two independent reviewers will screen abstracts of all citations that meet study eligibility in the first screen as outlined above. The second screen will consist of any studies selected by either one these reviewers. In the second screen, two independent reviewers will screen abstracts and full texts and agree on final study eligibility. The final number of articles to be obtained will be agreed upon with any disagreements on citations being resolved by consensus or by contacting a third reviewer who will serve as the tie breaker. Articles considered eligible after full-text review by the two independent reviewers will be included in the final set of studies for inclusion and those ineligible excluded from the final analysis. A log of all studies excluded and reasons for exclusion after the 1^st^ and 2^nd^ screen will be kept to account for any differences in inferences made.

The two reviewers will independently extract data using a standardized preformed data extraction form, the data compared and any discrepancies will be resolved by consensus. The abstracted data will be entered into a database for analysis.

**Data Items:**

We will abstract data on the study population including age, parity, and severity of malaria, drug exposures, and pregnancy outcomes. For a complete list of variables to be abstracted, see the data abstraction tools.

Outcomes: We will abstract information on all adverse pregnancy outcomes including miscarriage (pregnancy loss before 28 weeks), stillbirth (pregnancy loss at or after 28 weeks), and congenital anomalies (major and minor). We will equally prioritize each of the three adverse pregnancy outcomes.

**Quality Assessment:**

We will assess the quality of the clinical trials using The Cochrane Collaboration’s tool for assessing risk of bias. Five domains of bias tackling selection (sequence generation and allocation concealment), performance (blinding of participants and personnel; and other potential threats to validity), detection (blinding of outcome assessment; and other potential threats to validity), attrition (incomplete outcome data), and reporting (selective outcome reporting) bias will be assessed. We will assess bias in cohort studies using the Newcastle Ottawa scale that evaluates studies for selection bias, comparability, and assessment of the outcome.

**Data Analysis**

We will conduct meta-analyses to generate pooled estimates of the event rate and relative risk with 95% confidence intervals (CI) for miscarriage, stillbirth and congenital anomalies stratified by study type. Given that safety outcomes are rare outcomes, we may calculate pooled risk differences instead of pooled relative risks in order to account for zero cells. To fully characterize the risk, we will compare women exposed to any artemisinins to women exposed to SP, quinine, and chloroquine, and to no antimalarial drug (and therefore no malaria). We will use random effects models and measure the amount of heterogeneity present in the data evaluated by the I^2^ values. Because the importance of the observed value of the I^2^ value depends on both the magnitude and direction of the effect as well the strength of evidence of heterogeneity from statistical testing, we will roughly use the following categories of the I^2^:  0% to 40%: might not be important; 30% to 60%: may represent moderate heterogeneity; 50% to 90%: may represent substantial heterogeneity; 75% to 100%: considerable heterogeneity. We will conduct stratified analyses by study type, geographic location, trimester of exposure, and study time period. We will conduct sensitivity analyses to assess the influence of study quality on the results. Additionally we will assess publication bias through funnel plots. A two-tailed test p value of < 0.05 will be considered statistically significant.

As a secondary analysis, using only the randomized controlled trials, we will use meta-analyses to estimate the risk of stillbirth, miscarriage, and congenital anomalies using direct and indirect comparisons of different specific Artemisinin Combination Therapies (ACT) to each and other, and to non-artemisinin based therapies.

**References:**

1. Dellicour S, Hall S, Chandramohan D, Greenwood B. The safety of artemisinins during pregnancy : a pressing question. Malar J. 2007;10:1–10.

2. Dellicour S, Tatem AJ, Guerra CA, Snow RW, ter Kuile FO. Quantifying the number of pregnancies at risk of malaria in 2007: a demographic study. PLoS Med. 2010 Jan;7(1):e1000221.

3. Desai M, ter Kuile FO, Nosten F, McGready R, Asamoa K, Brabin B, et al. Epidemiology and burden of malaria in pregnancy. Lancet Infect Dis. 2007 Feb;7(2):93–104.

4. World Health Organization. World Malaria Report 2012. 2012.

5. Nosten F, McGready R, d’Alessandro U, Bonell A, Verhoeff F, Menendez C, et al. Antimalarial drugs in pregnancy: a review. Curr Drug Saf. 2006 Jan;1(1):1–15.

6. White NJ, McGready RM, Nosten FH. New medicines for tropical diseases in pregnancy: catch-22. PLoS Med. 2008 Jun 17;5(6):e133.

7. Li Q, Weina PJ. Severe embryotoxicity of artemisinin derivatives in experimental animals, but possibly safe in pregnant women. Molecules. 2010 Jan;15(1):40–57.

8. Clark RL. Embryotoxicity of the artemisinin antimalarials and potential consequences for use in women in the first trimester. Reprod Toxicol. Elsevier Inc.; 2009 Nov;28(3):285–96.

9. Piola P, Nabasumba C, Turyakira E, Dhorda M, Lindegardh N, Nyehangane D, et al. Efficacy and safety of artemether-lumefantrine compared with quinine in pregnant women with uncomplicated Plasmodium falciparum malaria: an open-label, randomised, non-inferiority trial. Lancet Infect Dis. Elsevier Ltd; 2010 Nov;10(11):762–9.

10. Rulisa S, Kaligirwa N, Agaba S, Karema C, Mens PF, de Vries PJ. Pharmacovigilance of artemether-lumefantrine in pregnant women followed until delivery in Rwanda. Malar J; 2012 Jan;11(1):225.

11. Manyando C, Mkandawire R, Puma L, Sinkala M, Mpabalwani E, Njunju E, et al. Safety of artemether-lumefantrine in pregnant women with malaria: results of a prospective cohort study in Zambia. Malar J. 2010 Jan;9:249.

12. McGready R, Lee SJ, J W, Ashley AE, Rijken MJ, Boel M, et al. Adverse eff ects of falciparum and vivax malaria and the safety of antimalarial treatment in early pregnancy : a population-based study. Lancet Infect Dis. 2012;12(May).

13. Mcgready R, Tan SO, Ashley EA, Pimanpanarak M, Viladpai-nguen J, Barends M, et al. A Randomised Controlled Trial of Artemether-Lumefantrine Versus Artesunate for Uncomplicated Plasmodium falciparum Treatment in Pregnancy. PLoS Med. 2008;5(12).

14. McGready R, Brockman a, Cho T, Cho D, van Vugt M, Luxemburger C, et al. Randomized comparison of mefloquine-artesunate versus quinine in the treatment of multidrug-resistant falciparum malaria in pregnancy. Trans R Soc Trop Med Hyg. 2000;94(6):689–93.

15. McGready R, Cho T, Keo NK, Thwai KL, Villegas L, Looareesuwan S, et al. Artemisinin antimalarials in pregnancy: a prospective treatment study of 539 episodes of multidrug-resistant Plasmodium falciparum. Clin Infect Dis. 2001 Dec 15;33(12):2009–16.

16. Van Eijk AM, Hill J, Povall S, Reynolds A, Wong H, Ter Kuile FO. The Malaria in Pregnancy Library: a bibliometric review. Malar J. Malaria Journal; 2012 Jan;11(1):362.

| **SCREENING 1 *(Abstract)*** Study Screening ID    A  **e.g.A0001** | |
| --- | --- |
| 1 | First author: ________________ Publication Year: |
| 2 | Study design?  □RCT □ Prospective Cohort □ Other □Can’t tell |
| 3 | Study population includes pregnant women? □Yes □No □Can’t tell |
| 4 | Study population includes patients exposed to artemisinins?  □ Yes □No □Can’t tell |
| 5 | Reports on adverse pregnancy outcomes (miscarriage, stillbirth, congenital anomalies)?  □Yes □No □Can’t tell |
| **Eligibility Criteria** *Check all that apply* | |
| □ Study design is a clinical trial or prospective cohort  □ Study participants include pregnant women  □ Study participants exposed to artemisinins  □ Reports adverse pregnancy outcomes (stillbirths, miscarriages, congenital anomalies)  □ Can’t tell any of the above | |
| **Study Eligible?**  □ Yes □Can’t tell *Proceed to Study Screening 2*  □No …… *END HERE* | |
| Date  Form completed by _____(Initials)  ^dd mmm yyyy^ | |

| **SCREENING 2 *(Full Text Review)*** Study Screening ID    B  **e.g.B0001** | |
| --- | --- |
| 1 | First author: __________________ Publication Year: |
| 2 | Language: □English □Other: _______­­­­_____________ |
| 3 | Study design?  □RCT □ Prospective Cohort □ Other □Can’t tell |
| 4 | Study population includes pregnant women? □Yes □No |
| 5 | Study population exposed to an artemisinin? □Yes □No |
|  | Study reports severe adverse event? □Yes □No |
| **Eligibility Criteria** *Check all that apply* | |
| □ Full text in English  □ Study design is a clinical trial or prospective cohort  □ Study participants include pregnant women  □ Study participants exposed to an artemisinin  □ Study reports on severe adverse events | |
| **Study Eligible?**  □Yes *Proceed to DATA EXTRACTION SRF* ***(final analysis n_i_)***  □No …… *END HERE* ***(exclude from final analysis n_e_)*** | |
| Date  Form completed by _____(Initials)  ^dd mmm yyyy^ | |

| **C. DATA EXTRACTION SRF *(Full Analysis)*** Study Screening ID    C  **e.g.C0001** | |
| --- | --- |
| 1 | First author: __________________ Publication Year: |
| 2 | Country/Countries: _Thailand___________; ­­­­­­­­­­­­­­­­­­­­­_________________; ____________ |
| 3 | Malaria transmission intensity  □ Low □ Moderate □ High |
|  | Malaria parasites in circulation  □ *P. falciparum* □ *P. vivax* □ XBoth |
| 4 | Study start: MonthYear □Not documented |
| 5 | Study end: MonthYear □Not documented |
| 6 | Study Design:  □RCT □ Prospective cohort |
| 6 | Intervention 1:  Artemisinin _______________ Dose ________mg Days__________  Combination Drug_________________ Dose________ Days_________  Purpose:  □ Treatment □ IPT □ IST  Intervention 2:  Artemisinin _______________ Dose ________mg Days__________  Combination Drug_________________ Dose________ Days_________  Purpose:  □ Treatment □ IPT □ IST  Intervention 3:  Artemisinin _______________ Dose ________mg Days__________  Combination Drug_________________ Dose________ Days_________  Purpose:  □ Treatment □ IPT □ IST |
| 7 | Control:  Drug: ________________  Dose____________ Days_____________  Purpose:  □ Treatment □ IPT □ IST |
|  | Length of follow-up:  ______________ years months days (circle 1) |
| **Study Population** | |
|  | Inclusion criteria: |
|  | Exclusion criteria: |
|  | Measurement of gestational age by:  □ Ultrasound □ LMP □ Ballard Score □ Fundal height □ Not reported  □ Other _________ |
| 1 | Age in years:. □Mean □Median □Not documented  Age SD  Age range ____________________ |
| 2 | Age:  Number <5: Proportion % □ NR  Number 5-18: Proportion % □ NR  Adults: Proportion % □ NR  Pregnant women: Proportion % □ NR |
| 3 | Sex:  Number male: Proportion %  Number female: Proportion % |
| 4 | Patient factors by Intervention:   \|  \| Intervention 1:  _________  N (%) \| Intervention 2:  __________  N (%) \| Intervention 3:  _______  N (%) \| Control Arm  ___________  N (%) \| Total  N(%) \| \| --- \| --- \| --- \| --- \| --- \| --- \| \| Age <5 \|  \|  \|  \|  \|  \| \| Age 5-18 \|  \|  \|  \|  \|  \| \| Adults (or age>18) \|  \|  \|  \|  \|  \| \| Male \|  \|  \|  \|  \|  \| \| Female \|  \|  \|  \|  \|  \| \| Pregnant \|  \|  \|  \|  \|  \| \| Mean Parasetemia \|  \|  \|  \|  \|  \| \| Uncomplicated malaria \|  \|  \|  \|  \|  \| \| Severe malaria \|  \|  \|  \|  \|  \| \| Parity </=2 \|  \|  \|  \|  \|  \| \| Parity >2 \|  \|  \|  \|  \|  \| \| 1^st^ Trimester \|  \|  \|  \|  \|  \| \| 2^nd^ Trimester \|  \|  \|  \|  \|  \| \| 3^rd^ Trimester \|  \|  \|  \|  \|  \| |
| **Primary outcomes of interest** | |
| 1 | How many total SAEs were reported ?____________________   \|  \| Interv. 1:  N (%) \| Interv. 2  N (%) Art 1^st^ tri \| Interv. 3  N (%) \| Control arm  N (%) \| Total  N (%) \| \| --- \| --- \| --- \| --- \| --- \| --- \| \| Any SAE \|  \|  \|  \|  \|  \| \| Death \|  \|  \|  \|  \|  \| \| Hospitalizations \|  \|  \|  \|  \|  \| \| Stillbirth \|  \|  \|  \|  \|  \| \| Miscarriage \|  \|  \|  \|  \|  \| \| All Congenital Anomaly \|  \|  \|  \|  \|  \| \| CA1  ___________ \|  \|  \|  \|  \|  \| \| CA2  ____________ \|  \|  \|  \|  \|  \| \| CA3  _____________ \|  \|  \|  \|  \|  \| \| CA4 _____________ \|  \|  \|  \|  \|  \| \| CA5 _____________ \|  \|  \|  \|  \|  \| \| Early Neonatal Death (<7 days) \|  \|  \|  \|  \|  \|   How many SAEs were reported after 1^st^ trimester exposures?____________________   \|  \| Interv. 1:  N (%) \| Interv. 2  N (%) \| Interv. 3  N (%) \| Control arm  N (%) \| Total  N (%) \| \| --- \| --- \| --- \| --- \| --- \| --- \| \| Any SAE \|  \|  \|  \|  \|  \| \| Death \|  \|  \|  \|  \|  \| \| Hospitalizations \|  \|  \|  \|  \|  \| \| Stillbirth \|  \|  \|  \|  \|  \| \| Miscarriage \|  \|  \|  \|  \|  \| \| All Congenital Anomaly \|  \|  \|  \|  \|  \| \| CA1  ___________ \|  \|  \|  \|  \|  \| \| CA2  ____________ \|  \|  \|  \|  \|  \| \| CA3  _____________ \|  \|  \|  \|  \|  \| \| CA4 _____________ \|  \|  \|  \|  \|  \| \| CA5 _____________ \|  \|  \|  \|  \|  \| \| Early Neonatal Death (<7 days) \|  \|  \|  \|  \|  \|   Effect Estimates:  Exposure ____________________ Comparator _____________________  Stillbirth: ________________ OR RR RD (circle 1)    _________________ aOR aRR aRD (circle 1)  Variables adjusted for ________________________________________________________  Miscarriage: ________________ OR RR RD (circle 1)  _________________ aOR aRR aRD (circle 1)  Variables adjusted for ________________________________________________________  Congenital Anomalies: ________________ OR RR RD(circle 1)  _________________ aOR aRR aRD (circle 1)  Variables adjusted for ________________________________________________________ |
|  | Comments: |
| **Trial Quality:** The Cochrane Collaboration’s tool for assessing risk of bias. | |
| 1 | SELECTION BIAS   \|  \| Description \| Bias Judgment (Yes/No/unclear) \| \| --- \| --- \| --- \| \| RANDOM SEQUENCE GENERATION \|  \|  \| \| ALLOCATION CONCEALMENT \|  \|  \| |
| 2 | PERFOMANCE BIAS   \|  \| Description \| Bias Judgment (Yes/No/unclear) \| \| --- \| --- \| --- \| \| BLINDING OF PARTICIPANTS AND PERSONNEL \|  \|  \| |
| 3 | DETECTION BIAS   \|  \| Description \| Bias Judgment (Yes/No/unclear) \| \| --- \| --- \| --- \| \| BLINDING OF OUTCOME ASSESSMENT \|  \|  \| |
| 4 | ATTRITION BIAS   \|  \| Description \| Bias Judgment (Yes/No/unclear) \| \| --- \| --- \| --- \| \| INCOMPLETE OUTCOME DATA \|  \|  \|  \| |
| Date  Form completed by ________(Initials)  ^dd mmm yyyy^ | |

**Bias Assessment for Cohort Studies:**

| **Assessment of quality of a cohort study – Newcastle Ottawa Scale** |  |
| --- | --- |
| **Selection** (tick one box in each section) |  |
| 1. Representativeness of the intervention cohorta) truly representative of the average, pregnant, community-dwelling resident ★b) somewhat representative of the average, pregnant, community-dwelling resident ★c) selected group of patients, e.g. only certain socio-economic groups/areasd) no description of the derivation of the cohort | 🞏  🞏  🞏  🞏 |
| 2. Selection of the non intervention cohorta) drawn from the same community as the intervention cohort ★b) drawn from a different sourcec) no description of the derivation of the non intervention cohort | 🞏  🞏  🞏 |
| 3. Ascertainment of interventiona) secure record (eg health care record) ★b) structured interview ★c) written self reportd) other / no description | 🞏  🞏  🞏  🞏 |
| 4. Demonstration that outcome of interest was not present at start of studya) yes ★b) no | 🞏  🞏 |
| **Comparability** (tick one or both boxes, as appropriate) |  |
| 1. Comparability of cohorts on the basis of the design or analysisa) study controls for age and parity ★b) study controls for any additional factors (malaria severity, concomitant therapies) ★ | 🞏  🞏 |
| **Outcome** (tick one box in each section) |  |
| 1. Assessment of outcomea) independent blind assessment ★b) record linkage ★c) self report d) other / no description | 🞏  🞏  🞏  🞏 |
| 2. Was follow up long enough for outcomes to occura) yes, if median duration of follow-up >= 6 month ★b) no, if median duration of follow-up < 6 months | 🞏  🞏 |
| 3. Adequacy of follow up of cohortsa) complete follow up: all subjects accounted for ★b) subjects lost to follow up unlikely to introduce bias: number lost <= 20%, ★ or description of those lost suggesting no different from those followedc) follow up rate < 80% (select an adequate %) and no description of those lostd) no statement | 🞏  🞏  🞏  🞏 |
