# Supplementary material for: The Safety of Artemisinin Derivatives for the Treatment of Malaria in the 2nd or 3rd Trimester of Pregnancy: A Systematic Review and Meta-Analysis
Source: PLoS One. 2016 Nov 8;11(11):e0164963. doi: 10.1371/journal.pone.0164963 (PMC5100961; doi:10.1371/journal.pone.0164963)
Supplement: S1 Supplementary Methods — (DOCX) [file pone.0164963.s006.docx]

**The safety of the artemisinin antimalarials in pregnancy: a systematic review and meta-analysis of prospective cohort studies and randomized controlled trials**

**Supplementary Materials:**

**Methods search:**

A multi-concept Boolean search strategy was developed with the assistance of a librarian using keywords and MeSH terms with no restriction for time of publication or language. The search terms for Medline consisted of: (Pregnant women OR pregnan* AND malaria) AND (Artemisinin* OR “Artemisinin Combination Therapy” OR ACT OR artemether OR artesunate OR dihydroartemisinin OR treatment) AND (Pregnancy complication [mh] OR safety OR “serious adverse event” OR miscarriage OR stillbirth OR “pregnancy loss” OR “spontaneous abortion” OR “birth defect” OR congenital abnormalities OR “congenital malformations” OR “congenital anomalies”) AND Clinical trials OR trials OR cohort study [mh] OR prospective [tw]. Search terms for the other databases are included below:

**EMBASE Search:** January 13, 2015: 295 Articles

'pregnant woman' AND malaria AND (artemisinin* OR 'artemisinin combination therapy' OR act OR artemether OR artesunate OR dihydroartemisinin) AND [embase]/lim NOT [medline]/lim AND 'human'/de

**Malaria in Pregnancy Library:** January 13, 2015: 384 Articles

Artemisinin OR artemether OR artesunate OR dihydroartemisinin

**Supplementary Figures:**

**Supplementary Figure 1: PICOTS Framework of the systematic search**

**Supplementary Figure 2: PubMed Search Strategy, Search Date June 15, 2015**

**Supplementary Figure 3: Pooled Risk Difference for fetal loss after 2-3^rd^ trimester exposures to artemisinins, stratified by comparison group**

*McGready 2001 reported multiple types of artemisinin exposures were combined for this analysis and reported as artemisinins (12)

^McGready MQ or Q exposures include patients given MQ, Q, or both

ART: artesunate, AS-MQ: artesunate mefloquine, AL: artemether-lumefantrine, DP: dihydroartemisinin-piperaquine, AAP: artesunate atovaquone proguanil, AS-SP: artesunate sulfadoxine pyrimethamine, AS-AQ: artesunate-amodiaquine, CD: Chlorproguanil-dapsone; Q: quinine, Q+C: quinine+clindamycin, SP: sulfadoxine pyrimethamine, AQ: amodiaquine, SP+CQ: sulfadoxine pyrimethamine chloroquine, MQ: mefloquine, SP-AZM: sulfadoxine pyrimethamine azithromycin, Comm: community controls, IPT: intermittent preventative treatment, ACT: artemisinin combination therapy

References:

1. Adam I, Elwasila E, Mohammed Ali DA, Elansari E, Elbashir MI. Artemether in the treatment of falciparum malaria during pregnancy in eastern Sudan. Trans R Soc Trop Med Hyg. 2004;98(9):509–13.

2. Adam I, Ali DM, Abdalla M a. Artesunate plus sulfadoxine-pyrimethamine in the treatment of uncomplicated Plasmodium falciparum malaria during pregnancy in eastern Sudan. Trans R Soc Trop Med Hyg. 2006;100(7):632–5.

3. Deen JL, von Seidlein L, Pinder M, Walraven GE, Greenwood BM. The safety of the combination artesunate and pyrimethamine-sulfadoxine given during pregnancy. Trans R Soc Trop Med Hyg. 2001;95(4):424–8.

4. Manyando C, Mkandawire R, Puma L, Sinkala M, Mpabalwani E, Njunju E, et al. Safety of artemether-lumefantrine in pregnant women with malaria: results of a prospective cohort study in Zambia. Malar J. 2010 Jan;9:249.

5. McGready R, Cho T, Keo NK, Thwai KL, Villegas L, Looareesuwan S, et al. Artemisinin antimalarials in pregnancy: a prospective treatment study of 539 episodes of multidrug-resistant Plasmodium falciparum. Clin Infect Dis. 2001;33(12):2009–16.

6. McGready R, Nosten F. The Thai-Burmese border: drug studies of Plasmodium falciparum in pregnancy. Ann Trop Med Parasitol. 1999;93 Suppl 1(1):S19–23.

7. Mosha D, Guidi M, Mwingira F, Abdulla S, Mercier T, Decosterd LA, et al. Population Pharmacokinetics and Clinical Response for Artemether-Lumefantrine in Pregnant and Nonpregnant Women with Uncomplicated Plasmodium falciparum Malaria in Tanzania. Antimicrob Agents Chemother. 2014 Aug;58(8):4583–92.

8. Rulisa S, Kaligirwa N, Agaba S, Karema C, Mens PF, de Vries PJ. Pharmacovigilance of artemether-lumefantrine in pregnant women followed until delivery in Rwanda. Malar J.; 2012 Jan;11(1):225.

9. Poespoprodjo JR, Fobia W, Kenangalem E, Lampah D a, Sugiarto P, Tjitra E, et al. Dihydroartemisinin-piperaquine treatment of multidrug resistant falciparum and vivax malaria in pregnancy. PLoS One. 2014 Jan;9(1):e84976.

10. Wang T. Follow-up observation on the therapeutic effect and remote reactions of artemisinin (Qinghaosu) and artemether in treating malaria in pregnant women. J Tradit Chinese Med. 1989;9(1):28–30.

11. Nakelembe M, Kyabayinze D, Compaore Y, Gatton M, Hopkins H, Incardona S, et al. Detection of placental malaria and impact of rdt screening and treatment on pregnancy outcomes in areas of varied transmission. Am J Trop Med Hyg. M. Nakelembe, Department of Obstetrics and Gynaecology, School of Medicine, Makerere University, Kampala, Uganda; 2012;87(5):138.

12. Bounyasong S. Randomized Trial of Artesunate and Mefloquine in Comparison with Quinine Sulfate to Treat P. falciparum Malaria Pregnant Women. J Med Assoc Thail. 2001;84(9):1289–99.

13. Kalilani L, Mofolo I, Chaponda M, Rogerson SJ, Alker AP, Kwiek JJ, et al. A Randomized Controlled Pilot Trial of Azithromycin or Artesunate Added to Sulfadoxine-Pyrimethamine as Treatment for Malaria in Pregnant Women. 2007;(11).

14. McGready R, Brockman a, Cho T, Cho D, van Vugt M, Luxemburger C, et al. Randomized comparison of mefloquine-artesunate versus quinine in the treatment of multidrug-resistant falciparum malaria in pregnancy. Trans R Soc Trop Med Hyg. 2000;94(6):689–93.

15. McGready R, Cho T, Villegas L, Brockman A, Vi M Van, Looareesuwan S. Randomized comparison of quinine-clindamycin treatment of falciparum malaria in pregnancy versus attesunate in the. Trans R Soc Trop Med Hyg. 2001;95:651–6.

16. McGready R, Ashley EA, Moo E, Cho T, Barends M, Hutagalung R, et al. A Randomized Comparison of Artesunate- Atovaquone-Proguanil versus Quinine in Treatment for Uncomplicated Falciparum Malaria during Pregnancy. J Infect Dis. 2005;63110.

17. McGready R, Tan SO, Ashley EA, Pimanpanarak M, Viladpai-nguen J, Barends M, et al. A Randomised Controlled Trial of Artemether-Lumefantrine Versus Artesunate for Uncomplicated Plasmodium falciparum Treatment in Pregnancy. PLoS Med. 2008;5(12).

18. Mutabingwa TK, Muze K, Ord R, Briceño M, Greenwood BM, Drakeley C, et al. Randomized trial of artesunate+amodiaquine, sulfadoxine-pyrimethamine+amodiaquine, chlorproguanal-dapsone and SP for malaria in pregnancy in Tanzania. PLoS One. 2009 Jan;4(4):e5138.

19. Piola P, Nabasumba C, Turyakira E, Dhorda M, Lindegardh N, Nyehangane D, et al. Efficacy and safety of artemether-lumefantrine compared with quinine in pregnant women with uncomplicated Plasmodium falciparum malaria: an open-label, randomised, non-inferiority trial. Lancet Infect Dis. Elsevier Ltd; 2010 Nov;10(11):762–9.

20. Sowunmi a, Oduola a M, Ogundahunsi O a, Fehintola F a, Ilesanmi O a, Akinyinka OO, et al. Randomised trial of artemether versus artemether and mefloquine for the treatment of chloroquine/sufadoxine-pyrimethamine-resistant falciparum malaria during pregnancy. J Obstet Gynaecol. 1998;18(4):322–7.

21. Mcgready R, Kang J, Watts I, Tyrosvoutis MEG, Torchinsky MB, Htut AM, et al. Audit of antenatal screening for syphilis and HIV in migrant and refugee women on the Thai-Myanmar border : a descriptive study [ version 2 ; referees : 2 approved ] Referee Status : F1000Research. 2015;3(123):1–23.
