# Supplementary material for: The Safety of Artemisinin Derivatives for the Treatment of Malaria in the 2nd or 3rd Trimester of Pregnancy: A Systematic Review and Meta-Analysis
Source: PLoS One. 2016 Nov 8;11(11):e0164963. doi: 10.1371/journal.pone.0164963 (PMC5100961; doi:10.1371/journal.pone.0164963)
Supplement: S1 Table — (DOCX) [file pone.0164963.s007.docx]

**Supplementary Table 1: Clinical presentation of pregnant women upon enrollment from included studies**

| **First Author** | **Gestational Age at Enrollment in weeks** | **HIV prevalence (Country Prevalence)** | **Fever (>38 °C)** | **Parasitemia** |
| --- | --- | --- | --- | --- |
| Adam 2004 (1) | Artemether:27 (s.d. 7.3) | NR (Moderate) | NR | 8468 (525-34500)** |
| Adam 2006 (2) | 29.7 (range 16-36) | NR (Moderate) | Mean 38.1 (range 37.5-40)^φ^ | 7762 (3800-15250) ^§^ |
| Deen 2001 (3) | NR | NR (Low) | NR | NR |
| Manyando 2010 (4) | NR | ACT: 30% tested No exp.: 38% tested ~20% positive (High) | NR | NR |
| McGready, 2001 (5) | Artemisinins 24.6 (range 3-40) | NR (low) | 17% | NR |
| McGready 1999(6) | NR | NR (Low) | AS: 16.0% | AS: 814 (16-130385)** |
|  |  |  | Q: 4.4% | Q: 1078 (4-53719) |
|  |  |  | MQ: 5.0% | MQ: 1249 (53-43257) |
| Mosha 2014 (7) | AL: 27 (range 14-37) | NR (High) | AL: 37.1 (range 36-39)**^φ^** | 25280 (560-198,080)^ |
| Rulisa 2012 (8) | AL: 25.8 (95%CI 25.3-26.4), No drug: 28.5 (95%CI 28-29) | NR (Moderate) | NR | NR |
| Poespoprodjo 2014 (9) | DP: 38 (24-44) | NR (Low) | DP: 10.7% | NR |
|  | DP+iv ART:39 (20-43) |  | DP + iv ART: 9% |  |
|  | Q: 39 (34-40) |  | Oral Q: 28% |  |
|  | CQ+SP: 38 (21-41) |  | CQ+SP: 33% |  |
|  | No exp.: 39 (19-44) |  | No exp.: 5.2% |  |
| Wang 1989 (10) | NR | NR (Low) | NR | NR |
| Nakelembe 2012 (11) | NR | All HIV negative (High) | NR | NR |
| Bounyasong 2001 (12) | AS-MQ: 26.7 weeks | NR (Moderate) | NR | AS-MQ: 1330* |
|  | Q: 26.4 weeks |  |  | Q: 1313 |
| Kalilani 2007 (13) | AS-SP: 22 (IQR 20-23) | AS-SP: 7/33 | NR | AS-SP: 685.6 (120-4259)** |
|  | SP: 22 (IQR 20-24) | SP: 9/26 |  | SP:963 (180-22499) |
|  | SP+AZM:24 (IQR 20-24) | SP+AZM: 10/28 (High) |  | SP+AZM: 1183 (150-22499) |
| McGready 2000 (14) | AS-MQ: 24 (range 12-40) | NR (low) | AS-MQ: 15.2 | AS-MQ: 11651 (32-241127)** |
|  | Q: 24 (15-38) |  | Q: 20.4% | Q: 19086 (79-149386) |
| McGready 2001 (15) | AS7: 28 (range 16-40) | NR (low) | AS7: 14.1% | AS7: 9822 (16-93019)** |
|  | Q+C26 (range 16-40) |  | Q+CD: 21.8% | Q+CD: 11098 (16-109724) |
| McGready 2005 (16) | AAP: 21 (s.d 5.3) | NR (low) | AAP: 59% | AAP: 2596 (33-123027)** |
|  | Q: 21 (s.d. 4.5) |  | Q: 73.8% | Q: 2083 (33-109648) |
| McGready 2008 (17) | AL: 23.7 (s.d. 6.8) | NR (low) | AL: 57.6% | AL: 3548 (48-158489)** |
|  | Artesunate: 24.8 (s.d. 7.6) |  | AS7: 58.6% | AS7: 3162 (65-457-088) |
| Mutabingwa 2009 (18) | AS-AQ: 6 months (IQR 5-8 months) | SP: 0/27 | NR | SP: 184 (55-535)* |
|  | SP: 7 months (IQR 6-8) | CD: 1/80 |  | CD: 106 (23-650) |
|  | SP-AQ: 7 (IQR 6-8) | SP+AQ: 1/82 |  | SP+AQ: 25 (51-578) |
|  | CD: 6 months (IQR 5-8) | AS+AQ: 0/79 (High) |  | AS+AQ: 181 (62-628) |
| Piola 2010 (19) | AL: 22.3 (range 9-38) | NR (High) | AL: 23% | AL: 1418 (IQR 4727)^§^ |
|  | Q: 24.7 (range 10-39) |  | Q: 20% | Q: 1995(IQR 9771) |
| Sowunmi 1998 (20) | NR | NR (Moderate) | AL: 38.1+/-0.2^ϕ^ | AL: 29719 (1674-250138)** |
|  |  |  | A+MQ: 38.1 +/-0.1 | A+MQ: 28044 (2001-199992) |

HIV prevalence low (<1%), moderate (1-5%), high (>5%) from the UNAIDS 2013 report, except for studies along the Thai/Burma border which were estimated using McGready et al. 2015 (21).

^Parasitemia (counts/µL)

*Median parasite count (per 200 WBC)

** Geometric mean density (range)

^§^Geometric mean density (IQR)

^φ^Mean temperature (°C)

Abbreviations: NR not reported, IQR interquartile range, AS-MQ artesunate mefloquine, AL artemether-lumefantrine, DP dihydroartemisinin-piperaquine, AAP artesunate- Atovaquone/Proguanil, AS-SP artesunate sulfadoxine pyrimethamine, AS-AQ artesunate-amodiaquine, CD: chlorproguanal-dapsone, Q quinine, Q+C quinine+clindamycin, SP sulfadoxine pyrimethamine, AQ amodiaquine, SP+CQ sulfadoxine pyrimethamine chloroquine, MQ mefloquine, SP-AZM sulfadoxine pyrimethamine azithromycin, No exp. No exposure, IPT intermittent preventative treatment, RCT randomized controlled trial, ACT artemisinin combination therapy, sd standard deviation
