# Supplementary material for: The Safety of Artemisinin Derivatives for the Treatment of Malaria in the 2nd or 3rd Trimester of Pregnancy: A Systematic Review and Meta-Analysis
Source: PLoS One. 2016 Nov 8;11(11):e0164963. doi: 10.1371/journal.pone.0164963 (PMC5100961; doi:10.1371/journal.pone.0164963)
Supplement: S2 Table — (DOCX) [file pone.0164963.s008.docx]

**Supplementary Table 2: Sensitivity analyses of pooled odds ratios using different modeling techniques and correction factors**

|  | M-H fixed with 0.5 corr. POR (95%CI) | D-L Random Effects with 0.5 corr. POR (95%CI) | Peto with 0.5 corr. POR (95%CI) | M-H fixed with 0.01 corr. POR (95%CI) | M-H fixed with 0.69 corr. POR (95%CI) |
| --- | --- | --- | --- | --- | --- |
| **2nd trimester exposures and risk of miscarriage** | |  |  |  |  |
| ACT vs. No exp. | 1.13 (0.77, 1.66) I^2^+86.7%, 3 studies | 1.59 (0.23, 10.33) | 1.13 (0.76-1.66) | 1.13 (0.77, 1.66) | 1.13 (0.77, 1.66) |
| **2 or 3rd trimester exposures and risk of congenital anomalies** | |  |  |  |  |
| ACT vs. Quinine | 1.00 (0.27, 3.75) I^2^=0%, 3 studies | 1.04 (0.26, 4.10) | 1.00 (0.25, 4.03) | 1.00 (0.25, 4.05) | 1.00 (0.27, 3.65) |
| ACT vs. No exp. | 0.79 (0.37, 1.67) I^2^=0%, 3 studies | 0.82 (0.36, 1.85) | 0.81 (0.41, 1.61) | 0.79 (0.37, 1.67) | 0.79 (0.37, 1.67) |
| ACT vs. SP | 1.37 (0.76, 2.39) I^2^=70.3%, 2 studies | 0.86 (0.18, 4.18) | 1.37 (0.76, 2.41) | 1.37 (0.78, 2.39) | 1.37 (0.78, 2.39) |
| **2 or 3rd trimester exposures and risk of stillbirth** | |  |  |  |  |
| ACT vs. Quinine | 0.49 (0.24, 0.97) I^2^=0%, 3 studies | 0.48 (0.24, 0.97) | 0.48 (0.24, 0.96) | 0.49 (0.24, 0.97) | 0.49 (0.24, 0.97) |
| ACT vs. No exp. | 1.10 (0.79, 1.54) I^2^=0%, 4 studies | 1.10 (0.79, 1.54) | 1.10 (0.79, 1.54) | 1.10 (0.79, 1.54) | 1.10 (0.79, 1.54) |
| ACT vs. SP | 0.93 (0.43, 2.01) I^2^=56.0%, 2 studies | 1.29 (0.23, 7.11) | 0.93 (0.43, 2.01) | 0.93 (0.43, 2.01) | 0.93 (0.43, 2.01) |
| ACT vs. SP combinations | 3.47 (0.46-26.03) I^2^=78.1%, 2 studies | 1.32 (0.01, 27.05) | 8.39 (1.20, 58.79) | 155.19 (0.0, 7.66e+07) | 2.62 (0.45, 15.02) |
| **2 or 3rd trimester exposures and risk of fetal loss** | |  |  |  |  |
| ACT vs. Quinine | 0.58 (0.31, 1.08) I^2^=0%, 6 studies | 0.56 (0.30, 1.04) | 0.57 (0.31, 1.06) | 0.58 (0.32, 1.08) | 0.58 (0.32, 1.08) |
| ACT vs. SP | 5.86 (0.73, 46.88) I^2^=0% 2 studies | 5.69 (0.70, 45.97) | 5.91 (1.31, 26.64) | 2.60 (0.0, 256e+08) | 4.43 (0.73, 26.96) |
| ACT vs. SP combinations | 1.71 (0.57, 5.07) I^2^=0%, 3 studies | 1.62 (0.53, 4.96) | 1.88 (0.67, 5.27) | 2.04 (0.65, 6.42) | 1.61 (0.55, 4.67) |

M-H Mantel Haenszel, D-L DerSimonian-Laird, ACT Artemisinin combination therapy, Corr continuity correction factor, SP sulfadoxine pyrimethamine, No exp. No exposure

*Random effects with DerSimonian-Laird models take into account the heterogeneity in the intervention effects of the studies being pooled. Peto Method uses an observed compared to expected model to create a “Peto OR,” but should be used cautiously when there are large differences in sizes of the comparison groups. Continuity correction factors can have a strong effect on studies based on their sample sizes, therefore we conducted sensitivity analyses using different correction factors.
