# Supplementary material for: The Safety of Artemisinin Derivatives for the Treatment of Malaria in the 2nd or 3rd Trimester of Pregnancy: A Systematic Review and Meta-Analysis
Source: PLoS One. 2016 Nov 8;11(11):e0164963. doi: 10.1371/journal.pone.0164963 (PMC5100961; doi:10.1371/journal.pone.0164963)
Supplement: S3 Table — (DOCX) [file pone.0164963.s009.docx]

**Supplementary Table 3: Newcastle Ottawa Scale Assessing Bias in Cohort Studies**

| Study | Selection (★★★★) | Comparability (★★) | Outcome (★★★) | Bias |
| --- | --- | --- | --- | --- |
| Adam 2004 | ★★ |  | ★★ | Moderate |
| Adam 2006 | ★★ |  | ★★ | Moderate |
| Dean 2001 | ★★★★ | ★ | ★★ | Low |
| Manyando 2010 | ★★★ | ★ | ★★★ | Low |
| McGready 2001 | ★★★ |  | ★★★ | Moderate |
| McGready 1999 | ★★ |  | ★★★ | Moderate |
| Mosha 2014 | ★★ |  | ★ | High |
| Rulisa 2012 | ★★★★ | ★ | ★★★ | Low |
| Poespoprodjo 2014 | ★★★ | ★ | ★★ | Moderate |
| Wang 1989 | ★★★ |  | ★★★ | Moderate |
| Nakelembe 2012 | ★★★★ |  | ★★ | Moderate |

The maximum number of stars for selection, comparability and outcome is 4, 2, and 3 respectively. Scores of 0-3, 4-6, and 7-9 were rated as high, moderate and low bias respectively.
