# Supplementary material for: The Safety of Artemisinin Derivatives for the Treatment of Malaria in the 2nd or 3rd Trimester of Pregnancy: A Systematic Review and Meta-Analysis
Source: PLoS One. 2016 Nov 8;11(11):e0164963. doi: 10.1371/journal.pone.0164963 (PMC5100961; doi:10.1371/journal.pone.0164963)
Supplement: S4 Table — (DOCX) [file pone.0164963.s010.docx]

**Supplementary Table 4: Cochrane Bias Assessment of Randomized Controlled Trials**

| **Study** | **Random Sequence Generation** | **Allocation Concealment** | **Blinding of participants and personnel** | **Blinding of outcome assessment** | **Incomplete outcome data** | **Overall threat of bias** |
| --- | --- | --- | --- | --- | --- | --- |
| Bounyasong, 2001 | **?** | **High** | **High** | **High** | **?** | **High** |
| Kalilani, 2007 | **Low** | **Low** | **High** | **High** | **?** | **Moderate** |
| McGready, 2000 | **?** | **Low** | **High** | **High** | **?** | **Moderate** |
| McGready, 2001 | **?** | **Low** | **High** | **High** | **?** | **High** |
| McGready, 2005 | **Low** | **Low** | **High** | **High** | **?** | **Moderate** |
| McGready, 2008 | **?** | **Low** | **High** | **High** | **Low** | **Moderate** |
| Mutabingwa, 2009 | **Low** | **Low** | **?** | **High** | **Low** | **Low** |
| Piola, 2010 | **Low** | **low** | **Low** | **?** | **High** | **Low** |
| Sowunmi, 1998 | **?** | **?** | **High** | **High** | **Low** | **Moderate** |
